# Late Holocene relative sea-level records from coral microatolls in Singapore

**DOI:** 10.1038/s41598-024-62937-9

**Published:** 2024-06-12

**Authors:** Fangyi Tan, Benjamin P. Horton, Lin Ke, Tanghua Li, Jennifer Quye-Sawyer, Joanne T. Y. Lim, Dongju Peng, Zihan Aw, Shi Jun Wee, Jing Ying Yeo, Ivan Haigh, Xianfeng Wang, Lin Thu Aung, Andrew Mitchell, Gina Sarkawi, Xinnan Li, Nurul Syafiqah Tan, Aron J. Meltzner

**Affiliations:** 1 Earth Observatory of Singapore, Nanyang Technological University, 50 Nanyang Avenue, Singapore, 639798 Singapore; 2 Asian School of the Environment, Nanyang Technological University, 50 Nanyang Avenue, Singapore, 639798 Singapore; 3 School of Ocean and Earth Science, University of Southampton, National Oceanography Centre, European Way, Southampton, SO14 3ZH UK

**Keywords:** Palaeoclimate, Palaeoceanography

## Abstract

Late Holocene relative sea-level (RSL) data are important to understand the drivers of RSL change, but there is a lack of precise RSL records from the Sunda Shelf. Here, we produced a Late Holocene RSL reconstruction from coral microatolls in Singapore, demonstrating for the first time the utility of *Diploastrea heliopora* microatolls as sea-level indicators. We produced 12 sea-level index points and three marine limiting data with a precision of < ± 0.2 m (2σ) and < ± 26 years uncertainties (95% highest density region). The data show a RSL fall of 0.31 ± 0.18 m between 2.8 and 0.6 thousand years before present (kyr BP), at rates between − 0.1 ± 0.3 and − 0.2 ± 0.7 mm/year. Surface profiles of the fossil coral microatolls suggest fluctuations in the rate of RSL fall: (1) stable between 2.8 and 2.5 kyr BP; (2) rising at ~ 1.8 kyr BP; and (3) stable from 0.8 to 0.6 kyr BP. The microatoll record shows general agreement with published, high-quality RSL data within the Sunda Shelf. Comparison to a suite of glacial isostatic adjustment (GIA) models indicate preference for lower viscosities in the mantle. However, more high quality and precise Late Holocene RSL data are needed to further evaluate the drivers of RSL change in the region and better constrain GIA model parameters.

## Introduction

Understanding the links between climate and relative sea level (RSL) in the Late Holocene (last 4000 years) provides context for future sea-level changes^1,2^. Late Holocene RSL reconstructions have been shown to track temperature changes within the last three millennia^2,3^. Late Holocene RSL proxy data have also been augmented with tide gauge data to study the time of emergence of modern rates of sea-level rise above pre-industrial background levels^4,5^. As processes such as glacial isostatic adjustment (GIA), ocean dynamics, tectonics and sediment compaction can result in local to regional RSL departures from global mean sea level, studies of the global time of emergence and global sea-level variability in the Late Holocene require a global distribution of RSL records^2,5^. However, the existing global Common Era sea-level databases are based heavily on data from the North Atlantic^2,3,5^.

Despite notable attempts to study Late Holocene RSL changes in the Sunda Shelf^6–8^, data inconsistencies and large uncertainties in the data make deciphering the drivers of RSL change in the region challenging^9^. The range of RSL elevations from proxy data in the Malay-Thai Peninsula at any time over the past ~ 3000 years is 3–4 m^10^, while the variability in RSL predicted by GIA models over this time period can reach up to ~ 3 m^11^ within the region, presenting challenges for tuning GIA models. Furthermore, some data from the Sunda Shelf suggest RSL of up to 3.4 m below present between ~ 3 and ~ 0.5 thousand years before present (kyr BP)^7,12,13^ while others do not^8,14^. Indeed, the published Singapore record exhibits a RSL lowstand in the Late Holocene^10^. However, there are only four sea-level index points (SLIPs) in the past ~ 3000 years, all from peats and muds, in part due to the lack of accommodation space for coastal wetland formation as RSL fell from the highstand^15^. These data points have RSL uncertainties of ± 1 m or more^10^ due to the large tidal range of Singapore.

**Figure 1:**
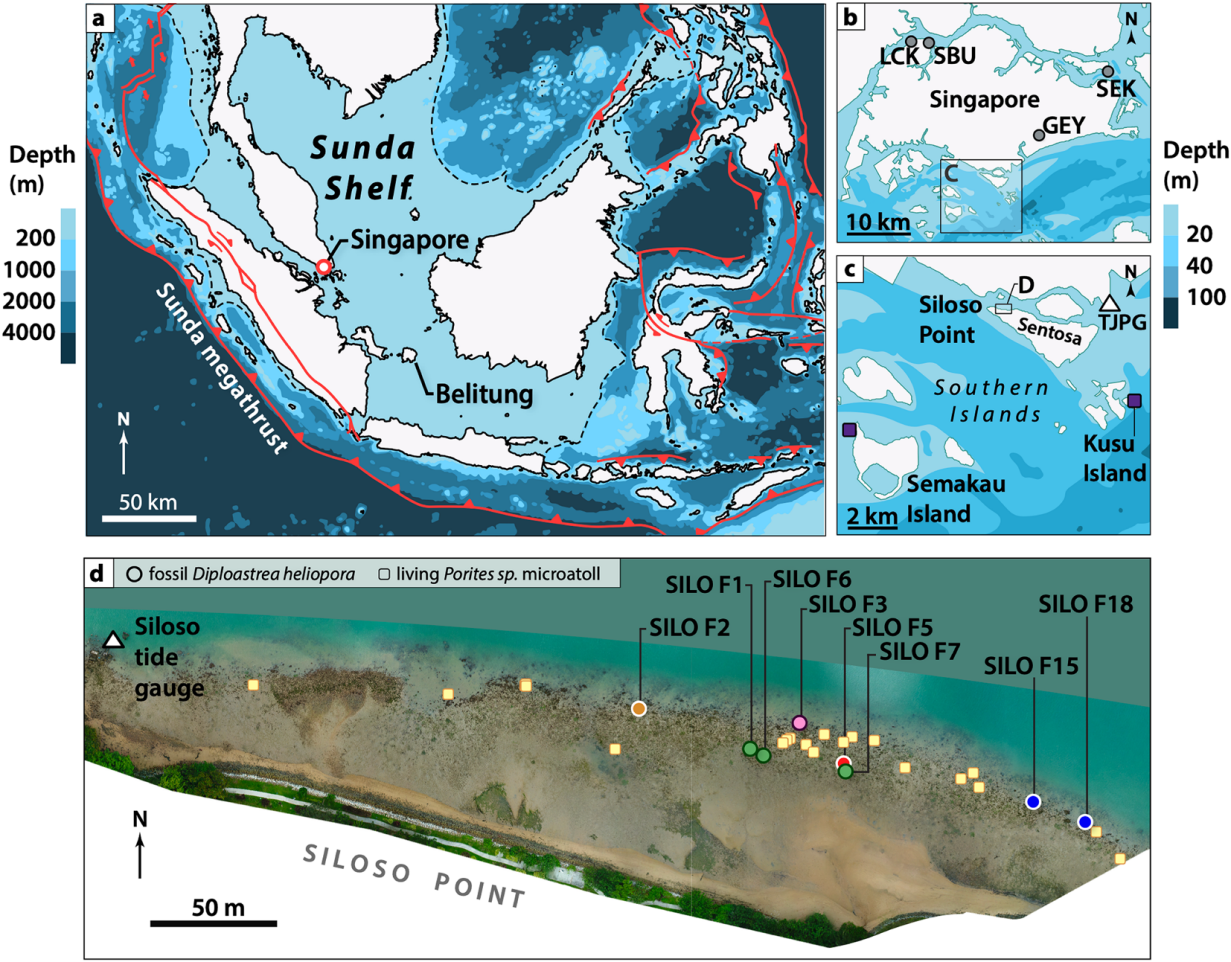
Locations of microatolls in this study. (**a**) Map of the study region. The boundary of the Sunda Shelf is based on the 200 m bathymetric contour^79^. Tectonic faults (red lines) are based on Refs.^43,80,81^. Bathymetry was made with Natural Earth. (**b**, **c**) Location of the Siloso Point reef in relation to (**b**) existing RSL data in Singapore^10^ (grey circles) and (**c**) living *Diploastrea heliopora* microatolls sites (dark purple squares) in the Southern Islands of Singapore. White triangle: Tanjong Pagar (TJPG) tide gauge managed by the Maritime Port Authority of Singapore (MPA). Bathymetry in panels b and c are adapted from Ref.^82^ LCK: Lim Chu Kang; SBU: Sungei Buloh; GEY: Geylang; SEK: Sekudu. (**d**) Orthomosaic of the Siloso Point reef, created using Agisoft Metashape 2.0.1 (https://www.agisoft.com/). Yellow squares: locations of living *Porites* sp*.* microatolls at Siloso Point. Filled circles: locations of fossil corals in this study, coloured by age groups. White triangle: tide gauge deployed in this study. All maps in this figure were generated using QGIS 3.32.3 (https://www.qgis.org/).

**Figure 2:**
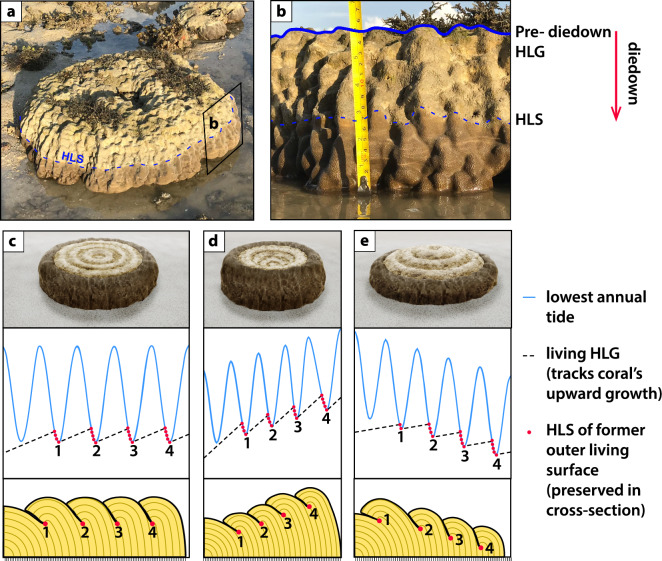
Coral microatoll growth tracks relative sea-level change. (**a**, **b**) Photographs of a living *Porites* sp*.* microatoll at Siloso Point, documented in year 2020 within 1–2 months of a ~ 7 cm diedown. (**c**–**e**) 3D schematic (top) and cross-sectional radial profiles (bottom) showing the coral microatoll HLG tracking (**c**) stable, (**d**) rising and (**e**) falling relative sea level. In (**c**)–(**e**), we show only every 4th annual band of each radial cross section for clarity. Each black tic mark at the bottom of (**c**)–(**e**) represents a year. The blue curves (middle) show changes in the lowest annual tide from year to year and represents the theoretical HLS that the coral can grow up to. Each time the coral HLG catches up to the lowest tide, a diedown occurs (red dot). Consecutive diedowns occur in years when the lowest annual tides get progressively lower. For simplicity, we label each cluster of diedowns collectively as one diedown (numbered); in the radial cross sections (**c**–**e**, bottom panels), we show only the lowest diedown in each cluster of diedowns for clarity. These labelled diedown clusters are ~ 18.61 years apart and are modulated by the lunar nodal cycle^38^. HLS: highest level of survival; HLG: highest level of growth. 3D coral microatoll schematics in (**c**)–(**e**) were developed using the 3D coral microatoll simulator of Ref.^83^.

**Figure 3:**
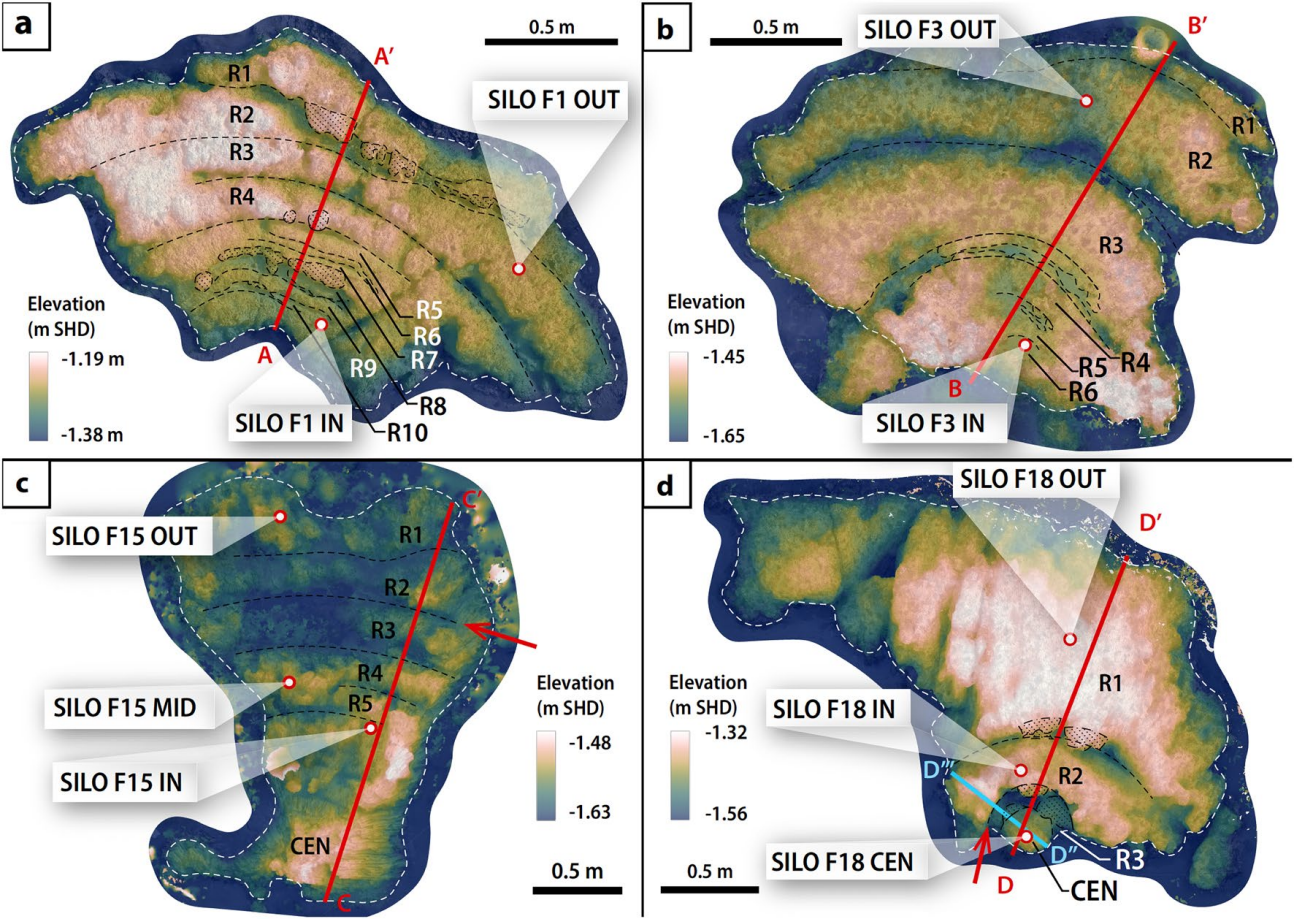
Digital surface models of selected fossil coral microatolls. Digital surface models overlain on orthomosaics of (**a**) SILO F1, (**b**) SILO F3, (**c**) SILO F15 and (**d**) SILO F18. Concentric rings are labelled from the youngest (outermost) to oldest (innermost), beginning with ring 1 (‘R1’); CEN: the inferred centre of the microatoll. Note that the ring labels are only internally consistent within each microatoll and rings with the same label do not indicate coeval features inferred across corals. Red and light blue lines: transects shown in Fig. 4 (A–Aʹ, B–Bʹ, C–Cʹ, D–Dʹ, D″–Dʹʹʹ); dashed black lines: inferred ring boundaries; dashed white lines: coral microatoll boundary; shaded regions: inferred overgrowth; white points bordered in red: core locations. Red arrows in (**c**) and (**d**) indicate the inferred diedown that is common to both SILO F15 and SILO F18.

Here, we present the first RSL reconstructions from fossil coral microatolls in Singapore—and, to our knowledge, the first RSL reconstruction to use *Diploastrea heliopora* microatolls globally (Fig. 1). Coral microatolls are fixed biological indicators whose upward growth is controlled by subaerial exposure at extreme low tides rather than accommodation space (Fig. 2)^16^. The coral microatolls in our study grow within a narrower indicative range than mangroves, supplying a vertical precision of < ± 0.2 m (2σ). ^230^Th dates provided a temporal precision of ≤ ± 26 years (95% highest density region; hereafter, HDR) in the RSL data. In addition, we used surface profiles of the microatolls to infer sea-level tendencies (i.e., whether RSL rose or fell over time) and to splice two overlapping coral microatoll records (Fig. 3). Our coral microatoll reconstruction shows good agreement with independent RSL records in an updated, quality-controlled database of Late Holocene RSL data from the Sunda Shelf. The fossil coral data add to the scarce RSL record in the Late Holocene for Singapore and offer an opportunity to detect centennial-scale fluctuations in RSL that previously could not be resolved given the larger uncertainties and inconsistencies of the existing data. Importantly, the Singapore coral microatoll data indicate preference for GIA models that incorporate lower mantle viscosities, illustrating the importance of the record for GIA model validation.

## Study area

Singapore is located ~ 700 km from the Sunda megathrust, in the core of the Sunda Shelf that is thought to be tectonically stable, with low rates of internal deformation^17,18^ (Fig. 1a). Most bedrock faults in Singapore are presumed to have been inactive since the Neogene^19^, although there is some evidence to suggest slight faulting during the Neogene or Quaternary within the Bedok Formation^19^. Modern-day coseismic subsidence of ~ 2 cm^20^ and post-seismic subsidence of ~ 2–6 mm/year have been recorded by tide gauges and Global Navigation Satellite System (GNSS) measurements in Singapore following the 2004 Sunda megathrust earthquake in Sumatra^21,22^.

RSL data since the Last Glacial Maximum (LGM) from the Sunda Shelf suggest rates of sea-level changes have been variable^23^. Rates of RSL rise doubled from up to 7.0 ± 5.8 mm/year after the LGM (~ 20 kyr BP) to as much as 15.4 ± 8.2 mm/year during meltwater-pulse 1A (between ~ 14.7 and ~ 14.3 kyr BP)^24,25^. This rapid RSL rise flooded the Sunda Shelf and severed the land bridge connecting Singapore to the Riau Islands by ~ 8 kyr BP^25^. During the early Holocene, there was a slowdown in the rate of RSL rise between ~ 8.5 kyr BP and 7 kyr BP, associated with the final stages of melting of major ice sheets^10,12^. This RSL rise was followed by mid-Holocene highstands of variable timing and magnitude within the region, before a Late Holocene RSL fall^11^. Some studies suggest the presence of a Late Holocene RSL lowstand between 1 and 3.4 m below present at ~ 1 kyr BP^6,7,12,13^. In the twentieth and twenty-first centuries, rates of RSL rise in the vicinity of Singapore increased from 0.0 ± 1.6 mm/year (2σ) between 1915 and 1990 to 1.0 ± 2.1 mm/year (2σ) between 1990 and 2019^26^.

Our study site is located at Siloso Point near the northwestern tip of Sentosa (1.2594° N, 103.8122° E), in the Southern Islands of Singapore (Fig. 1). The site is a narrow, free-draining reef ~ 450 m long and ~ 80 m wide (Fig. 1d), underlain by Upper Triassic interbedded sandstones and mudstones of the Fort Siloso Formation^27^. The Fort Siloso Formation at the study site is offset to the west compared to southern extensions of the same formation along an unnamed fault^19^. A southwest-dipping thrust fault terminates east of the study site^19^.

The bedrock geology controls the reef substrate. Fossil *Diploastrea heliopora* microatolls are interspersed with living microatolls (primarily *Porites* sp*.*) (Fig. S1) and small isolated scleractinian coral heads near the edge of the reef, which is composed mainly of sandy substrate. Patches of the reef closer to the mudstones are underlain predominantly by mud, and here the reef is colonised by the green algae *Halimeda* sp. All corals in this study are located within ~ 30 m of the edge of the reef, beyond which the reef drops off to depths of more than 0.8 m below mean low water spring (MLWS) tide. There are no structures suggesting any former ponding along this narrow reef, which could bias the RSL reconstructions high^28^. Spring tidal range based on our portable tide gauge sensor at the site (23 July 2020 to 15 August 2022) is 2.6 m (Supporting Text S1), similar to that recorded by the nearby Tanjong Pagar Tide Gauge (1.2617° N, 103.8517° E)^29^ (Fig. 1c & Fig. S2).

## Results

### Coral elevations

At Siloso Point, we found living *Porites* sp*.* microatolls between lowest astronomical tide (LAT) and mean low water spring tide (MLWS) (Fig. S7). The weighted mean highest level of growth (HLG) of *Porites* sp. microatolls at the site is − 1.42 ± 0.04 m SHD or 0.20 ± 0.04 m above LAT (2σ, standard error of the weighted mean, n = 24).

No living *Diploastrea heliopora* microatolls were discovered at Siloso Point, but a systematic inter-genus difference between the living HLG of *Diploastrea heliopora* and *Porites* sp*.* microatolls was observed at the nearby Kusu and Semakau Islands (Fig. 1c and Fig. S7). Application of this inter-genus difference to the living HLG of *Porites* sp. microatolls at Siloso Point derived the indicative meaning of *Diploastrea heliopora* microatolls at Siloso Point, which was used to quantify past RSL. The indicative meaning comprises two components: (1) the indicative range (± 0.10 m, 2σ) and (2) its central tendency, the reference water level (− 1.51 m SHD) (Supporting Texts S6 and S7).

**Figure 4:**
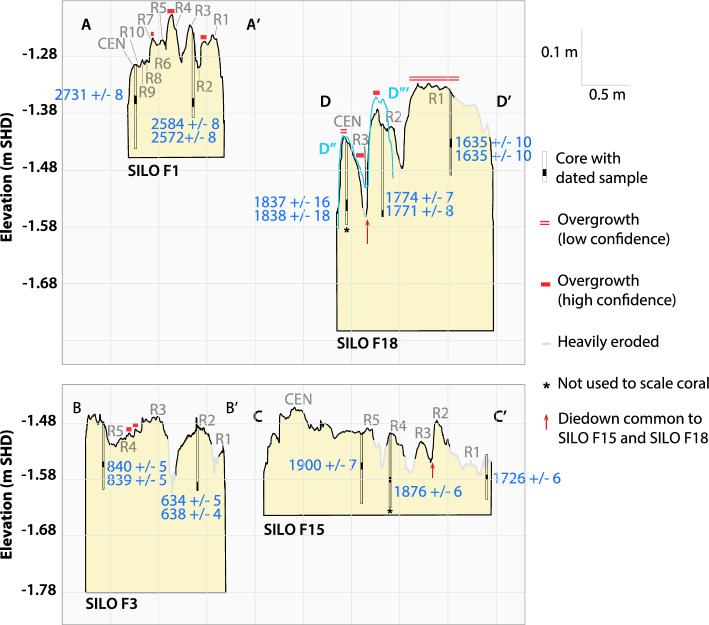
U-Th dates from coral cores are in sequence (ages become younger going radially outwards). Radial cross-sectional profiles of fossil *Diploastrea heliopora* coral microatolls SILO F1, SILO F3, SILO F15 and SILO F18, indicating position of cores (hollow rectangles) and sample depths that were subsampled for dating (black rectangles). Blue numbers: U-Th ages (years BP). BP: “Before Present”, where present refers to the year 1950 CE. The “IN” and “OUT” cores in each microatoll (excluding those marked with an *; see Table 1) were used to scale each fossil coral microatoll from horizontal distance to age, to produce the cross-sectional profiles in Fig. 5B. Note that all four microatolls have been scaled to the same vertical and horizontal scales, and that the elevations are true to the y-axis, but the corals are not horizontally positioned in any given order. For SILO F18, a second radial transect (D″–Dʹʹʹ) is shown to illustrate R3, which is eroded in the main transect (D–Dʹ) (see Fig. 3).

Fossil *Diploastrea heliopora* corals were discovered at Siloso Point between − 1.5 and − 1.2 m SHD (Table 1, Fig. 4). SILO F1, SILO F2, SILO F5, SILO F6 and SILO F7 were the highest fossil corals (found between − 1.3 and − 1.2 m SHD), followed by SILO F18 (found at ~ − 1.4 m SHD). SILO F15 and SILO F3 were the lowest fossil corals at the site, found at − 1.5 m SHD.

**Table 1:** Ages and elevations of Siloso Point fossil corals. For details on the ^230^Th ages, refer to Supporting document SI1. HDR: highest density region.

Sample ID	Elevation of top of core (m SHD)	Sample depth (m)	^230^Th age of dated sample (year BP) (2σ)^a^	Extrapolated age of top of core (year BP) (95% HDR)
SILO F3 OUT	− 1.54	0.11	636 ± 3^b^	633 ± 4
SILO F3 IN	− 1.51	0.08	839 ± 3^b^	836 ± 5
SILO F18 OUT	− 1.33	0.09	1635 ± 7^b^	1634 ± 7
SILO F18 IN	− 1.40	0.16	1773 ± 5^b^	1754 ± 16
SILO F18 CEN	− 1.43	0.12	1837 ± 12^b^	1807 ± 21
SILO F15 OUT	− 1.54	0.06	1726 ± 6	1715 ± 13
SILO F15 MID	− 1.50	0.14	1876 ± 6	1853 ± 26
SILO F15 IN	− 1.49	0.09	1900 ± 7	1884 ± 18
SILO F2 OUT	− 1.23	0.10	2087 ± 6	2072 ± 10
SILO F2 IN	− 1.19	0.07	2685 ± 7^b^	2669 ± 11
SILO F5	− 1.22	0.08	2239 ± 7	2228 ± 10
SILO F1 OUT	− 1.26	0.12	2578 ± 6^b^	2570 ± 7
SILO F1 IN	− 1.30	0.06	2731 ± 8	2729 ± 8
SILO F6	− 1.20	0.07	2787 ± 9	2782 ± 10
SILO F7	− 1.23	0.08	2812 ± 10	2805 ± 12

^a^The ^230^Th ages have been corrected for initial detrital Thorium assuming an ^230^Th/^232^Th atomic ratio of 4.4 ± 2.2 × 10^−6^. Refer to Supporting Text S4 for details on the sensitivity test conducted on the assumed ^230^Th/^232^Th atomic ratio.

^b^Age here is the weighted mean age and standard error derived from subsamples of the same core.

### Coral chronology

The ages obtained from individual vertical coral cores were in sequence, with the inner cores returning older ages than the outer cores (Fig. 4 & Fig. S5, Table 1). All replicated subsamples (‘B1’ and ‘B2’ samples) yielded similar mean corrected ^230^Th ages that differed by less than 12 years between replicates (Supporting Document SI1, ‘U-series’ sheet), demonstrating reproducibility. All U-Th dated samples had initial δ^234^U values with 2σ uncertainties that fell within the 145 ± 5‰ range for modern seawater^30^, which suggests negligible open system behaviour (Supporting Document SI1). The ages of the RSL data, governed by the extrapolated ages of the core tops, range from ~ 2.8 to ~ 0.6 kyr BP, with uncertainties ranging from ± 4 to ± 26 years (95% HDR) (Table 2; Supporting Text S5).

**Table 2:** Relative sea level (RSL) from fossil *Diploastrea heliopora* corals at Siloso Point, Sentosa. Ages here are the modelled ages for the top of the core (refer to Table 1). HDR: highest density region.

Sample ID	Age (year BP) (95% HDR)	Age (CE) (95% HDR)	RSL (m) (2σ)	Type
SILO F3 OUT	633 ± 4	1317 ± 4	− 0.06 ± 0.13	Sea-level index point
SILO F3 IN	836 ± 5	1114 ± 5	− 0.03 ± 0.13	Sea-level index point
SILO F18 OUT	1634 ± 7	316 ± 7	0.15 ± 0.13	Sea-level index point
SILO F18 IN	1754 ± 16	196 ± 16	0.09 ± 0.13	Sea-level index point
SILO F18 CEN	1807 ± 21	143 ± 21	0.05 ± 0.13	Sea-level index point
SILO F15 OUT	1715 ± 13	235 ± 13	0.02 ± 0.16	Sea-level index point
SILO F15 MID	1853 ± 26	97 ± 26	0.06 ± 0.16	Sea-level index point
SILO F15 IN	1884 ± 18	66 ± 18	0.07 ± 0.16	Sea-level index point
SILO F2 OUT	2072 ± 10	− 122 ± 10	> 0.13	Marine limiting
SILO F2 IN	2669 ± 11	− 719 ± 11	> 0.17	Marine limiting
SILO F5	2228 ± 10	− 278 ± 10	> 0.14	Marine limiting
SILO F1 OUT	2570 ± 7	− 620 ± 7	0.23 ± 0.13	Sea-level index point
SILO F1 IN	2729 ± 8	− 779 ± 8	0.19 ± 0.13	Sea-level index point
SILO F6	2782 ± 10	− 832 ± 10	0.28 ± 0.13	Sea-level index point
SILO F7	2805 ± 12	− 855 ± 12	0.25 ± 0.13	Sea-level index point

**Figure 5:**
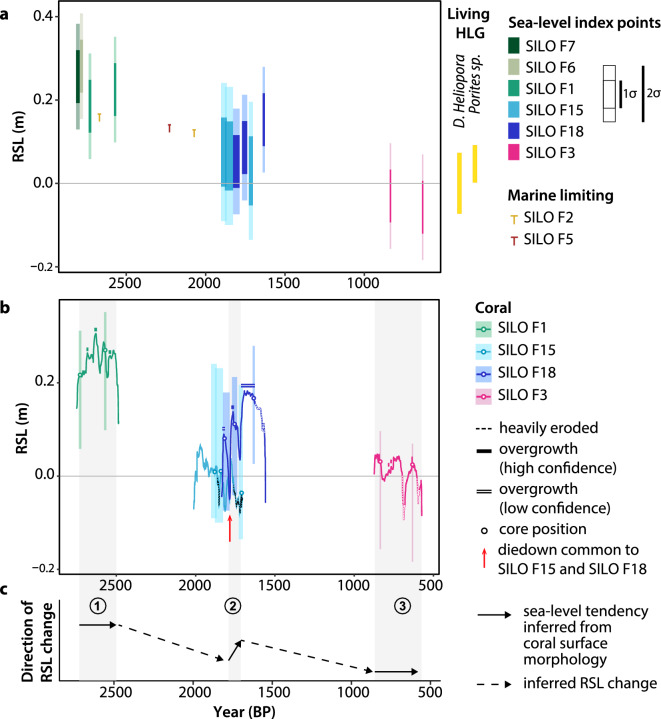
RSL record from fossil corals at Siloso Point, Sentosa, Singapore. (**a**) Sea-level index points (SLIPs) and marine limiting data points colour coded by coral. Yellow rectangles indicate the indicative range (2σ) of the living highest level of growth (HLG) measured and estimated accordingly for living *Porites* sp*.* and *Diploastrea heliopora* coral microatolls at Siloso Point, Sentosa, between 2020 and 2022*.* The horizontal line of the marine limiting data points are plotted at the bottom of the RSL uncertainty and indicate that RSL could have been anywhere at or above the horizontal line; the vertical tick marks are only symbolic and their lengths do not represent RSL uncertainty. (**b**) Radial cross-sectional profiles extracted from digital surface models (Figs. 3 & 4) and superimposed onto the SLIPs (coloured shaded boxes) for the dated cores. The cross-sectional profiles can be translated vertically and horizontally, but the position of each core (circle) must lie within its modelled age and elevation uncertainties (indicated by the bounds of the corresponding SLIP). (**c**) Direction of RSL change inferred from coral microatoll surface morphologies as sea-level tendencies (solid arrows) or from the relative elevations of successive SLIPs (dashed arrows). Shaded vertical grey bars and corresponding numbered labels (1, 2 and 3) indicate the three distinct periods when sea-level tendencies were inferred. RSL: relative sea level.

The coral microatolls cluster in three age ranges. The oldest microatolls cluster around 2.8–2.6 kyr BP (Fig. 5a, Table 2). They consist of three corals (SILO F1, SILO F6, SILO F7) that grew within proximity (between 4 and 40 m) of one another (Fig. 1d), and which had similar morphologies and elevations (Figs. S6 and S10). The second group of SLIPs cluster from 1.9 to 1.6 kyr BP (Fig. 5a, Table 2). This age cluster consists of two microatolls (SILO F15 and SILO F18) that also grew close to each other (Fig. 1d). The ages suggest that the corals grew coevally during parts of their lifetimes and should therefore have experienced a shared RSL history. The youngest SLIPs are from a single coral microatoll (SILO F3), with an age range of 0.8 to 0.6 kyr BP (Fig. 5a).

### Relative sea-level reconstruction at Siloso Point, Sentosa

We produced 12 new SLIPs and three marine limiting data points by comparing the elevations of the fossil *Diploastrea heliopora* corals to the reference water level of living *Diploastrea heliopora* microatolls at Siloso Point (Table 2, Supporting Document SI1). An additional uni-directional erosion uncertainty (+ 0.08 ± 0.11 m, 2σ) was determined for the SILO F15 SLIPs (Supporting Text S8).

**Figure 6:**
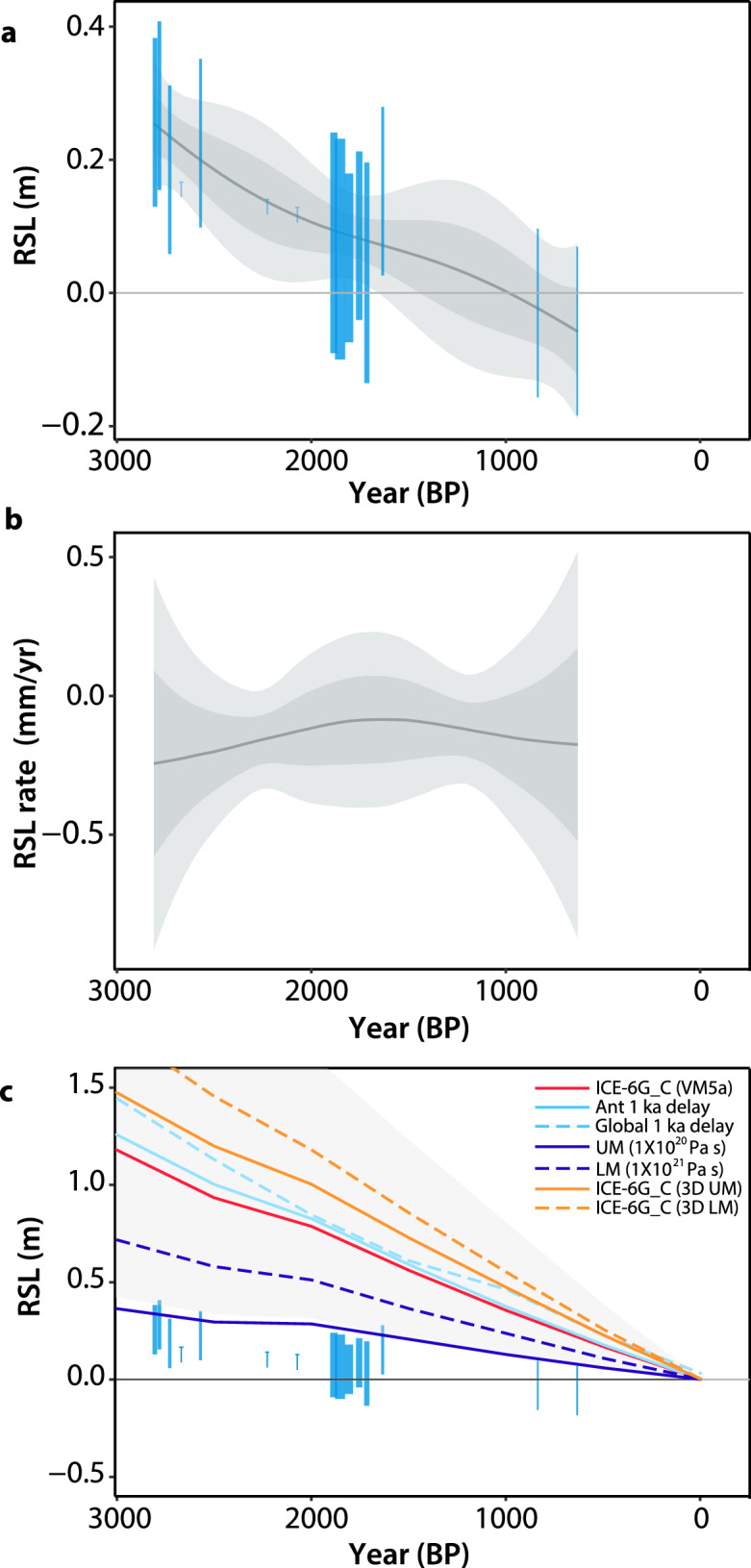
Magnitude and rates of RSL change compared to glacial isostatic adjustment models. (**a**) Magnitude of RSL and (**b**) rates of RSL change from fossil corals at Siloso Point, Sentosa, Singapore (blue). Grey curves indicate the mean, 1σ and 2σ range of the Errors-In-Variables Integrated Gaussian Process (EIV-IGP) model predictions^71^. In (**b**), the lower uncertainties at times corresponding to data gaps are an artifact of the model. (**c**) RSL data from Siloso Point compared to an ensemble of GIA model predictions^11^. The GIA model ensemble encompasses a variety of upper mantle (UM) and lower mantle (LM) viscosities, as well as ice-melting histories (global 1 kyr delay; 1 kyr delay in ice melting from Antarctica), modified with reference to the ICE-6G_C (VM5a) model^76^. Individual lines show selected GIA model predictions; the grey shaded wedge shows the 95% credible interval of the GIA model ensemble predictions. Rectangles: sea-level index points; T-shaped symbols: marine limiting data. The horizontal line of the T-shaped symbols is plotted at the bottom of the RSL uncertainty and indicate that RSL could have been anywhere at or above the horizontal line. The age axis is in years ‘before present’ (BP), where ‘present’ refers to the year 1950 CE. RSL: relative sea level.

The SLIPs at Siloso Point indicate a long-term RSL fall of 0.31 ± 0.18 mm between 2.8 kyr BP and 0.6 kyr BP (Fig. 6a), although short-lived (decadal to centennial) RSL excursions greater than ± 0.5 m cannot be precluded during the temporal gaps between 2.6 kyr BP and 1.9 kyr BP, and between 1.6 kyr BP and 0.8 kyr BP. Application of the EIV-IGP model suggests RSL fell at rates between 0.1 ± 0.3 mm/year and 0.2 ± 0.7 mm/year (2σ) (Fig. 6b). Two massive fossil corals (SILO F2 and SILO F5) did not have concentric rings preserved and were interpreted as marine limiting data (Fig. 5a). They show that RSL was at least 0.17 m above present at 2.7 kyr BP and at least 0.13 m to 0.14 m above present from 2.2 to 2.1 kyr BP.

Given the limited understanding of the indicative meaning of *Diploastrea heliopora* microatolls, an alternative reconstruction was produced to test the sensitivity of the Siloso Point record to a more conservative indicative meaning. Application of a wider indicative range (between LAT and midway between mean low water neaps (MLWN) and MLWS^28^) expands the lower bound of the SLIPs downwards by 0.35 m, whereas the upper bounds of the SLIPs remain mostly unchanged (Fig. S12; Supporting Document SI1). With the conservative indicative meaning, the Siloso Point SLIPs still indicate a Late Holocene RSL fall. The rates of RSL fall are similar (between 0.1 ± 0.6 and 0.2 ± 1.2 mm/year, 2σ) but with larger uncertainties (compared to between 0.1 ± 0.3 and 0.2 ± 0.7 mm/year, 2σ).

We supported our understanding of RSL changes with cross-sectional profiles of four coral microatolls with clear concentric-ringed structures (Figs. 3, 4 & 5b). Ignoring eroded segments of the profiles, the differences in HLG across the concentric rings of SILO F1 and SILO F3 are small (< 9 cm and < 6 cm, respectively; Fig. 4), showing they grew during periods of RSL stability (no tendency). The RSL stability inferred from the surface profiles of SILO F1 and SILO F3 are independently supported by the elevations of their SLIPs, which all overlap at the 2σ level (Fig. 5).

In contrast, SILO F18 has concentric rings that rise radially outwards, providing clear signs of RSL rise (positive sea-level tendency) in the earlier part of its lifetime, between 1.78 and 1.73 kyr BP (from R3 to R2). HLG on SILO F18 steps up radially outwards by 16 cm from R3 to R2, ignoring the higher parts of R2 that are interpreted as overgrowth (Fig. 4). In the later period between 1.73 and 1.70 kyr BP (from R2 to R1), HLG increases by less than or equal to 8 cm. The overgrowth inferred on R1 suggests there are more concentric rings hidden beneath the overgrowth that cannot be observed from the surface. While the presence of overgrowth itself must suggest that RSL had to have risen in the past to form overgrowth (Figs. 3d, 4, and Fig. S3), the timing of this rise and/or possibility of earlier periods with stable or falling RSL that were masked by the overgrowth cannot be precluded. Therefore, we cannot conclude on the sea-level tendencies during this time. The morphology of SILO F15 had been altered by erosion and similarly cannot supply information on sea-level tendencies (Supporting Text S8).

### Comparison with Sunda Shelf Late Holocene RSL database and GIA models

**Figure 7:**
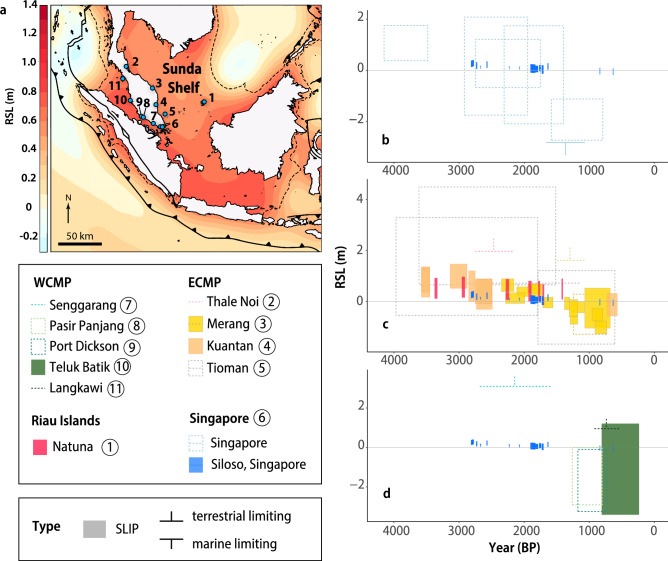
Map and regional comparison of RSL data. (**a**) Map of the study area showing the location of major faults^43,80^ and existing studies of Late Holocene RSL in the region (locations 1–11), created using QGIS 3.32.3 (https://www.qgis.org/). 1: Natuna Island^1,2^; 2: Thale Noi, Thailand^84^; 3: Merang, Terengganu^6,7^; 4: Kuantan^31^; 5: Tioman^14^; 6: Singapore^12,40,41^; 7: Senggarang^13^; 8: Pasir Panjang, Malaysia^13^; 9: Port Dickson^13^; 10: Teluk Batik^39^; 11: Langkawi^39^. The boundary of the Sunda Shelf (dashed line) is based on the 200 m bathymetric contour^79^. Background: map of the ICE-6G_C HetML140 glacial isostatic adjustment model^77,78^ at 2 kyr BP, generated using GMT 5.4.5 (https://www.generic-mapping-tools.org/). (**b**–**d**) Late Holocene RSL for Siloso Point, Sentosa, Singapore, compared to data from (**b**) elsewhere in Singapore (site 6); (**c**) East Coast Malay Peninsula (ECMP) and Riau Islands (sites 1–5); and (**d**) West Coast Malay Peninsula (WCMP) (sites 7–11). Dashed lines: low quality data; solid fill: high quality data. The horizontal line of marine limiting data is plotted at the bottom of the RSL uncertainty and indicate that RSL could have been anywhere at or above the horizontal line, vice versa for terrestrial limiting data, whose horizontal line is plotted at the top of the RSL uncertainty and indicate RSL is at or below it. The vertical ticks in the limiting data are purely symbolic and do not represent the magnitude of RSL uncertainty. RSL: relative sea level; SLIP: sea-level index point.

The Siloso Point reconstruction shows broad agreement with an updated Sunda Shelf Late Holocene RSL database (Supporting Text S11). The RSL data at Siloso Point fall within uncertainty of coeval high-quality SLIPs from the East Coast Malay-Thai Peninsula^7,31^ and Riau Islands^8^ (Fig. 7), except at 0.8 kyr BP when the SLIP from Siloso Point plots between 0.1 and 1.4 m above the SLIPs from Merang^7^ (or up to 1.3 m above the Merang SLIPs if we assume the conservative indicative meaning for *Diploastrea heliopora* microatolls of LAT to midway between MLWN and MLWS) (Fig. S12).

The Siloso Point SLIPs plot at the lower limit of the GIA model predictions, showing misfit with most models apart from modifications of the ICE-6G_C (VM5a) model that incorporate lower mantle viscosities (Fig. 6c). Decreasing the lower mantle viscosity from ~ 2.6 × 10^21^ Pa s to 1.0 × 10^21^ Pa s reduces the misfit by 50% to ~ 0.4 m at 2.8 kyr BP (from a misfit of ~ 0.8 m at 2.8 kyr BP with respect to the reference ICE-6G_C (VM5a) model). Decreasing the upper mantle viscosity from 5.0 × 10^20^ Pa s to 1.0 × 10^20^ Pa s improves the fit even more, producing GIA predictions that marginally match the SLIPs. In contrast, introducing 3D structures to the mantle and/or delays in the deglaciation histories worsened the Late Holocene misfit compared to the ICE-6G_C (VM5a) model (Fig. 6c).

## Discussion

### Coral microatolls as accurate and precise sea-level indicators

We demonstrate the utility of fossil *Diploastrea heliopora* coral microatolls as accurate and precise sea-level indicators. To our knowledge, *Diploastrea heliopora* microatolls have not been used in sea-level studies before; the use of *Porites* sp. coral microatolls is more common^32,33^. Existing studies of *Diploastrea heliopora* commonly revolve around paleoclimate^34,35^ or ecology^36,37^ and do not make reference to the coral microatoll morphology. The concordance between our record and independent high-quality data from other proxies in the region lends support for the validity of *Diploastrea heliopora* microatolls as sea-level indicators.

The high vertical precision (< ± 0.2 m, 2σ) of our coral microatoll SLIPs is comparable to other coral microatoll studies in the region, which have RSL uncertainties of between ± 0.1 and ± 0.4 m (2σ)^8,26,38^. Our coral reconstruction offers improved precision compared to RSL data from other indicators in the Sunda Shelf region (e.g., mangrove sediments, emerged oysters, shore platform), which have RSL uncertainties of ± 0.2 m to ± 2.3 m (2σ)^6,7,13,14,31,39^ (Fig. 7). In Singapore, the existing Late Holocene SLIPs from peats and muds have vertical uncertainties ranging from ± 0.7 to ± 1.9 m (2σ)^10,12,40,41^ (Fig. 7).

The *Diploastrea heliopora* microatolls in our study also produced RSL data with accurate and precise ages (< ± 26 years, 95% HDR). Other coral microatolls in the region that use ^230^Th dates have similarly small uncertainties of ± 10 years to ± 66 years (2σ)^8,42^. The age uncertainties of our fossil corals are smaller than the radiocarbon-dated Late Holocene RSL data in the region, which range from at least ~ ± 37 years to as much as ~ ± 1186 years (2σ) (Fig. 7).

We demonstrate the potential for the surface morphologies of coral microatolls to be used to produce continuous records of RSL and to detect more detailed changes in RSL that are not resolvable within the uncertainty of SLIPs. Traditionally, coral microatoll studies that produce continuous records of RSL rely on the matching of coeval diedowns observed in cross sections within coral microatoll slabs^43,44^. While logistical constraints prohibited the retrieval of microatoll slabs in our study, we were still able to combine the records from two microatolls (SILO F15 and SILO F18) by matching a ring boundary common to both corals (Figs. 3, 4 & 5b), guided by the tight constraints provided by the precision of the ^230^Th dates.

Similar ‘wiggle-matching’ of corals with overlapping ages has been done in paleo-environmental studies by matching the δ^18^O signatures of coeval corals^45^. However, to our knowledge, the use of surface morphologies to splice together coral records has yet to be applied to RSL reconstructions. We suggest that with well-preserved surface morphologies, this ‘wiggle-matching’ approach could be the solution to producing temporally precise, continuous RSL records, particularly when accurate ^230^Th ages are challenging to obtain due to the presence of relatively large amounts of non-radiogenic ^230^Th, open-system behaviour or diagenesis^46^. *Diploastrea heliopora* coral microatolls provide an added advantage in that their longevity and slow growth rates (~ 2–6 mm/year)^47^ enable longer continuous records of RSL compared to *Porites* sp. fossil corals of comparable sizes^34^.

We were not limited by the challenges that can typically hinder the use of coral microatolls as accurate and precise sea-level indicators, such as ponding and erosion^48^. RSL records from the living and fossil coral microatolls in our study are unlikely to be biased by ponding as the corals are distributed close to the edge of a narrow, free-draining reef, with no evidence of any former ramparts that could have acted as a sill to pond water landwards (Fig. 1d). The preservation of overgrowth (out-of-sequence growth that grew during the coral’s lifetime; Fig. S3) and defined concentric ridges across the fossil coral microatolls indicates limited erosion since the corals’ death, except for SILO F15, to which we have applied an erosion correction.

We also argue that any changes in tidal range over the Late Holocene at our site are likely to be small. Ref.^38^ modelled the LAT at Belitung Island (which is also located in the middle of the Sunda Shelf; Fig. 1a) to be less than 10 cm lower than present given a RSL of ~ + 2 m at ~ 7 kyr BP. Given that Late Holocene RSL at Siloso Point is within ± 0.7 m of present-day levels, the effects of changes in tidal range are likely to be smaller, and within error of our RSL reconstructions (which are < 0.2 m). Furthermore, unlike sedimentary indicators that may be subject to sediment compaction over time^49^, the coral microatolls in our study are not prone to significant lowering as they sit on a consolidated, sandy reef substrate. Given the similar elevations of similarly-aged coral microatolls (SILO F1, SILO F6 and SILO F7 in one generation; SILO F15 and SILO F18 in another generation; Table 1) and the position of all fossil coral microatolls along the edge of the reef, parallel to the reef edge, we infer that the fossil corals are in situ and have not been moved by waves or slumped substrate. While SILO F3 was the only fossil coral microatoll of its elevation and age, we did not find any evidence for tilting that would be suggestive of slumping. Additionally, the agreement of the SILO F3 SLIPs with SLIPs from Merang^6^ and Kuantan^31^ provide corroborating evidence for the validity of the SILO F3 SLIPs.

Nonetheless, there is still limited understanding of the indicative meaning of *Diploastrea heliopora* microatolls, in part due to the lower tidal elevation of living *Diploastrea heliopora* microatolls that makes them more challenging to locate in the field than their more commonly studied *Porites* sp*.* counterparts (Fig. 5a). Future research would benefit from an improved understanding of the indicative meaning of *Diploastrea heliopora* microatolls in the study region and elsewhere. *Diploastrea heliopora* corals have been documented throughout the Indo-Pacific^47^, including Singapore^37^ (Fig. S13). They are found to inhabit both lagoonal and more exposed, higher-energy reef settings in atoll islands^50,51^, but are also tolerant of high sedimentation rates and turbidity^52^. *Diploastrea heliopora* corals also have the ability to occupy both steep and gentle slopes and are resistant to being moved by waves, due to their firm attachment to the basal substrate^53^.

### Late Holocene RSL in the Sunda Shelf

We produced a new high-resolution Late Holocene RSL record from Singapore, which spans a time period when data from Singapore are lacking^10^ (Fig. 7). The SLIPs in our study indicate a net fall in RSL since 2.8 kyr BP, with long-term rates of RSL change between − 0.1 ± 0.3 mm/year and − 0.2 ± 0.7 mm/year (Fig. 6). A Late Holocene RSL fall from a highstand in equatorial locations such as Singapore is commonplace due to continental levering and ocean syphoning^11,54,55^. Analyses of the coral microatoll surface morphologies reveal higher-frequency RSL fluctuations in the Late Holocene in Singapore (Fig. 5b,c). Three periods with distinct sea-level tendencies were inferred: (1) stable RSL (no tendency) from 2.8 to 2.5 kyr BP; (2) RSL rise of 0.16 m at ~ 1.8 kyr BP (positive tendency) and (3) stable RSL from 0.8 to 0.6 kyr BP (no tendency).

We demonstrate the utility of the Siloso Point RSL record for GIA model validation. The SLIPs from Siloso Point lie mostly below the GIA model predictions—even with the use of a more conservative indicative meaning (Figs. S6c and S12)—and indicates preference for low upper mantle viscosities. Such preference for low upper mantle viscosities was similarly suggested by a previous GIA study using RSL data from far field regions^56^. In contrast, incorporation of a 3D Earth structure in both the upper and lower mantle enlarges the data-model misfit. This might be because the 1D background viscosity within the reference VM5a (e.g., 5.0 × 10^20^ Pa s in the upper mantle) is too high, such that adding a 3D structure to it would deteriorate the fit (e.g., Fig. 4 of Ref.^57^).

Interestingly, delays in deglaciation histories did not improve model fit in the Late Holocene, as was suggested by published studies in the region for the early to mid Holocene^7,10,31^. Although delaying the ice melting reduces the magnitude of the mid-Holocene highstand^11^, it slightly enlarges the misfit with the Late Holocene SLIPs in this study (Fig. 6c). This indicates that a simple delay in the deglaciation history (i.e., ice-equivalent sea level) is insufficient, and refinements to the deglaciation rates of ice sheets are necessary to achieve better fit with the Late Holocene data^58^.

Given that the Late Holocene SLIPs at Siloso Point only marginally intersect the lower bound of the 2σ uncertainty range of the GIA model predictions from Ref.^11^, it is possible that the SLIPs were influenced by other local to regional (non-GIA) processes that would have shifted the SLIPs lower. Long-term subsidence has been suggested for the region, of between 0.06 and 0.19 mm/year since the beginning of the Last Interglacial^59^ and between 0.2 and 0.3 mm/year over the Pleistocene^60^. On shorter timescales, Ref.^61^ inferred modern (2014–2020) vertical land motion rates of between − 4 and 0.5 mm/year across Singapore using InSAR. However, it is unclear if such rates are influenced by far-field effects of seismic ruptures along the Sunda megathrust, and if so, how much of the far-field deformation is permanent^20,21^.

Previously, Ref.^9^ noted inconsistencies within data from the Malay-Thai Peninsula, highlighting the possibility of reworking and sediment compaction as reasons for the discrepancies between the data. While the updated Late Holocene RSL database largely resolves the data inconsistencies (Fig. 7), the presence and nature of a Late Holocene RSL lowstand in the Sunda Shelf remains elusive. High-quality SLIPs from East Coast Malay-Thai Peninsula^7^ suggest a RSL lowstand up to 1.3 m below present between 1.6 kyr BP and 0.9 kyr BP, although the 0.8 kyr BP SLIP from Siloso Point indicates RSL within ± 0.2 m of present-day levels (Fig. 7 & Fig. S12). Two SLIPs from mangrove sediments in West Coast Malay-Thai Peninsula^13^ and existing Late Holocene SLIPs from Singapore^12,40^, which were previously used to suggest regional Late Holocene lowstands up to 3 m below present^10^, are now classified as low quality due to the uncertain degree of post-depositional lowering from sediment compaction, possible age contamination, and a lack of evidence to support the provenance of the mangrove sediments (Supporting document SI1; Supporting Text S11). Due to the lack of high-quality SLIPs within the region during this time (Fig. 7), we cannot conclude on the presence and regional expression of a Late Holocene RSL lowstand. More high-quality and precise RSL records are needed to evaluate the spatial extent of the Late Holocene RSL lowstand, decipher the drivers of RSL change in the region, and better constrain GIA model parameters.

## Methods

### Coral microatolls as sea-level indicators

Coral microatolls are fixed biological indicators that grow within the lower intertidal zone^33^ (Fig. 2). The concentric rings of a microatoll are diagnostic features permitting its use as a sea-level indicator; where preserved, they indicate that the microatoll was growing within the lower intertidal zone and was intermittently exposed during extreme low tides^26^.

The HLG of the coral microatoll just before a diedown (pre-diedown HLG), or the elevation of the ring crest, provides a filtered record of RSL changes through time^62^ (Supporting Text S2). The HLGs of successive concentric rings also provide information about sea-level tendencies^63^. A sea-level tendency is traditionally applied to sedimentary indicators and describes an increase or decrease in marine influence^64,65^. Here, we use the terminology specifically to infer the direction of RSL change. Concentric rings (and therefore HLG) that rise radially outwards indicate RSL rise (positive sea-level tendency) over the coral’s lifetime, and vice versa, although out-of-sequence rings (termed ‘overgrowth’) can form during periods of rising RSL (Fig. 2 & Fig. S3). Successive concentric rings with similar HLG elevations show stable RSL, which we assign as having no sea-level tendency (Fig. 2).

We reconstruct RSL from the surface morphologies of the fossil coral microatolls. While traditional methods of slabbing provide greater detail of the RSL changes from year to year^38^, analyses of the coral microatoll surface, paired with dates from coral cores, provide sufficient temporal resolution for understanding Late Holocene RSL changes.

### Coral elevations

We surveyed the ring crests (pre-diedown HLG) on all fossil corals and the HLG or highest level of survival (HLS) of living coral microatolls using a total station or digital level (Supporting Text S3). Performing RSL calculations using the relative elevations of pre-diedown HLG avoids the uncertainties associated with the sensitivity of diedown magnitudes to non-tidal drivers of RSL (Supporting Text S2). All elevations were related to the tides and the national geodetic datum, the Singapore Height Datum (SHD) (Supporting Text S3). The elevations of the living microatolls were compared to tidal datums that were derived directly from the turning points of astronomical tides predicted over an 18.61-year period, corresponding to the lunar nodal cycle^66^ (Supporting Text S1).

### Coral chronology

We drilled two to three vertical (~ 15 cm long, ~ 2 cm diameter) cores each from several fossil coral microatolls with concentric rings (SILO F1, SILO F3, SILO F15 and SILO F18) and one to two cores each from fossil corals that were more eroded (SILO F2, SILO F5, SILO F6 and SILO F7) (Fig. 3).

We visually inspected the cores and subsampled the most pristine portions for ^230^Th dating, avoiding the discoloured upper sections of the cores^8^ (Table 1, Fig. 4 & Fig. S4). All samples were pre-screened for calcite using powder X-ray diffraction (XRD) and dated using a Neptune Plus multi-collector inductively coupled plasma mass spectrometer (MC-ICP-MS) (Supporting Text S4). As subsequent RSL calculations are based on the relative elevations of the ring crests, a core-specific age extrapolation was made to the ^230^Th ages to derive the ages of the ring crests (top of the coral cores) based on the sample depth, growth angles and growth rates (Table 1, Supporting Text S5, Figs. S4 & S5). Some of the extrapolated age distributions for the core tops are not normally distributed, so we use the 95% credible interval range of the HDR of the ^230^Th age distributions (Table 1)^67^. Unless otherwise stated, all ages are expressed in years ‘BP’ (before present), where ‘present’ refers to the year 1950 CE.

### Sea-level index points and marine limiting data

We produced SLIPs from the measured ages and elevations of the fossil coral microatolls^8,68^ (Fig. 5). The age component of the SLIPs was derived from the 95% HDR of the extrapolated age of the core top (Table 1, Supporting Text S5, Fig. S5). The vertical component of a SLIP is described by an uncertainty (governed largely by the indicative range of the microatolls) about a central tendency (*RSL*_*j*_). The indicative range refers to the elevation range of the HLG of living microatolls, relative to the tides^8^. The midpoint of the indicative range is the reference water level^69^.

In coral microatoll studies, past RSL is commonly determined by comparing the elevation of the fossil coral microatolls to the reference water level of their living counterparts of the same genus at the same site (e.g., Refs.^63,68^). At Siloso Point, this was not possible as we did not discover any living equivalents of the fossil *Diploastrea heliopora* microatolls at the site. To overcome this, we applied an adjustment to derive the theoretical reference water level for *Diploastrea heliopora* microatolls (*E*_*dl*_) at Siloso Point. Different genera of coral microatolls can survive at different elevations due to differential tolerance to subaerial exposure and other environmental parameters^63,70^. Accordingly, the adjustment was calculated using observations of the relative elevations of living *Diploastrea heliopora* microatolls and *Porites* sp. microatolls at the nearby Kusu and Semakau Islands—and assumes that there is a systematic difference in the indicative meaning between *Diploastrea heliopora* and *Porites* sp*.* microatolls at any given site (Supporting Text S6, Fig. 1c).

The RSL indicated by each dated sample (or core) is estimated as follows:

1 $${RSL}_{j}= {E}_{j, df}-{E}_{dl}$$

where *E*_*j,df*_ is the surveyed surface elevation of the j-th coral core. An example of the RSL reconstruction is provided for SILO F3 OUT (Supporting Text S2).

To quantify the total vertical uncertainty (2σ) for each j-th sample (${\varepsilon }_{j,total}$), we added in quadrature all uncertainty sources (${\varepsilon }_{j,total}$) (Supporting Text S7):

2 $${\varepsilon }_{j,total }=\sqrt{{\varepsilon }_{j,1}^{2}+ {\varepsilon }_{j,2}^{2}+{\varepsilon }_{j,3}^{2}+{\varepsilon }_{j,4}^{2}+{\varepsilon }_{j,5}^{2}+{\varepsilon }_{j,6}^{2}}$$

Additional corrections were made to the RSL component of the SLIPs to account for the following: (1) a systematic offset in the elevations of living HLG, which were surveyed within a year or two after a diedown and more closely approximated HLS, rather than the pre-diedown HLG; and (2) significant erosion on SILO F15 (Supporting Text S8).

We applied the Errors-In-Variables Integrated Gaussian Process (EIV-IGP) model^71^ to the SLIPs to quantify rates of RSL change (Fig. 6). The EIV-IGP model is a Bayesian model that inverts magnitudes of RSL from the rates of RSL change and accounts for both the vertical and temporal uncertainties of the data. In this model, RSL is modelled as the integral of the RSL rate process using a Gaussian Process prior on the RSL rates. Temporal uncertainties are accounted for by adopting an Errors-In-Variables framework. We note that the EIV-IGP model does not model marine limiting data.

Fossil corals that do not have clear concentric rings preserved were used as marine limiting data^28^ (SILO F2 and SILO F5; Fig. S6). RSL for marine limiting data were calculated in the same way as SLIPs, but we represent marine limiting data as T-shaped symbols (Fig. 5a). To test the robustness of the Siloso Point RSL record, we additionally conducted a sensitivity test assuming a more conservative indicative meaning (between LAT and midway between MLWN and MLWS^28^) in RSL calculations (Supporting document SI1, Fig. S12).

### Continuous record of RSL

We used cross-sectional profiles of the coral microatolls to uncover sea-level tendencies. We constructed 3D digital surface models of the fossil microatolls SILO F1, SILO F15, SILO F18, and SILO F3 using Structure-from-Motion photogrammetry, processed in Agisoft Metashape (Fig. 3; Supporting Text S9). A cross-sectional elevation profile was extracted from each georeferenced digital surface model in QGIS along a radial transect that was selected to best represent the RSL history recorded by the microatoll: we selected the transect to capture the highest number of concentric rings possible while avoiding (to the extent possible) particularly eroded sections of the coral and areas of overgrowth (Fig. 3).

In our study, we expanded the photogrammetry method of Ref.^32^ to estimate not only the magnitude of sea-level changes, but to also produce continuous time-series from the radial transects. We translated the distance along each cross-sectional profile into age estimates using the horizontal (radial) distance between the inner and outer cores and the estimated age difference between the crests of the sampled rings (Fig. 4; Supporting Text S10). Each cross-sectional profile can be interpreted as a floating chronology that can be shifted to fit within the modelled age uncertainties of its cores (Fig. 5b).

We used the variations in HLG elevation across successive concentric rings on fossil coral microatolls to infer sea-level tendencies. The variability in living HLG observed around individual *Porites* sp. coral microatoll colonies is commonly < 5 cm^33,72^, but, to the best of our knowledge, no studies of the HLG variability in living *Diploastrea heliopora* microatolls have been conducted. We applied 15 cm as the threshold to determine sea-level tendencies as that is the largest HLG range observed on the living rim of a given living *Diploastrea heliopora* microatoll in our study (Supporting Document SI2), which we interpret as the natural variability in HLG that can be expected in the absence of any RSL change. HLG variability of < 15 cm was interpreted to indicate stable RSL (no tendency). Coral microatolls with HLG that increases (decreases) radially outwards by more than 15 cm were interpreted as rising (falling) RSL, with positive (negative) sea-level tendency.

### Updated Late Holocene RSL database and glacial isostatic adjustment modelling

To provide context for the Siloso Point coral microatoll record, we produced an updated Late Holocene RSL database for the interior of the Sunda Shelf following the HOLocene SEA-level variability (HOLSEA) database protocol^73^, which builds upon earlier regional databases^9,10^ (Supporting Text S11; Supporting Document SI1). All radiocarbon ages were standardised to use the latest IntCal20^74^ and Marine20^75^ calibration curves. We additionally assessed the quality of data based on their susceptibility to age and/or elevation errors^28^.

The Late Holocene SLIPs from Siloso Point were compared to an ensemble of GIA models (Fig. 6c). The GIA models comprise the widely used 1D model ICE-6G_C (VM5a)^76^ and other models modified from ICE-6G_C (VM5a), changing only one parameter of the ICE-6G_C VM5a each time. The modifications include decreases in the 1D upper and lower mantle viscosities, incorporation of a 3D Earth model^77,78^, and delays in the deglaciation histories, which were supported by previous studies from the region^7,10,31^. We also compared the SLIPs with the 2σ uncertainties of the GIA model ensemble predictions from Ref.^11^, considering GIA input parameters uncertainties. We did not conduct an iterative search for the optimal ice- and earth-model pairing as our data are restricted to the Late Holocene and provide only limited constraints on GIA models.

### Supplementary Information

Supplementary Information 1.

Supplementary Information 2.

Supplementary Information 3.

Supplementary Information 4.

## Data Availability

The data relevant to this study are openly available in the Nanyang Technological University data repository at 10.21979/N9/BRBZQC.
