# Supplementary material for: Late Holocene relative sea-level records from coral microatolls in Singapore
Source: Sci Rep. 2024 Jun 12;14:13458. doi: 10.1038/s41598-024-62937-9 (PMC11166974; doi:10.1038/s41598-024-62937-9)
Supplement: Supplementary file 1 — Supplementary Information 1. [file 41598_2024_62937_MOESM1_ESM.pdf]

Supporting Information for

Late Holocene relative sea-level records from coral microatolls in Singapore

Fangyi Tan[1,2] *, Benjamin P Horton[1,2], Lin Ke[1**], Tanghua Li[1**], Quye-Sawyer Jennifer[1,2], Joanne TY Lim[1,2], Dongju Peng[1], Zihan Aw[1,2], Shi Jun Wee[2], Jing Ying Yeo[1,2], Ivan Haigh[3], Xianfeng Wang[1,2], Lin Thu Aung[1], Andrew Mitchell[1,2], Gina Sarkawi[1,2], Xinnan Li[1], Nurul Syafiqah Tan[1,2], Aron J Meltzner[1,2]

[1] Earth Observatory of Singapore, Nanyang Technological University, 50 Nanyang Avenue, Singapore, 639798, Singapore

[2] Asian School of the Environment, Nanyang Technological University, 50 Nanyang Avenue, Singapore, 639798, Singapore

[3] School of Ocean and Earth Science, University of Southampton, National Oceanography Centre, European Way, Southampton, SO14 3ZH, UK

* Corresponding author: Fangyi Tan (fangyi001@e.ntu.edu.sg, fangyi.tan21@gmail.com)

** Both authors contributed equally

Contents of this file

Text S1 to S11

Figures S1 to S13

Tables S1 (within Text S4), S2 (within Text S7), S3 to S5 (within Text S11)

References

Acronyms

HLG: highest level of growth; HLS: highest level of survival; SLIP: sea-level index point; RSL: relative sea level

Text S1. Tidal levels at Siloso Point

Portable ONSET HOBO U20L pressure-sensor tide gauges were deployed at the site (1.26006°N, 103.80986°E) from 23 July 2020 to 15 August 2022. Each tide gauge deployment was calibrated to a physical measurement of water depth (measured with a tape measure from the sensor to the water surface) to correct for instrument drift over time (hereafter referred to as 'reference water level' measurements). To align and ground truth the tide gauge measurements, we also conducted measurements of tidal levels using a water level tube in the field (hereafter referred to as 'direct survey' measurements). Successive water level readings from the water level tube (taken every 5 seconds over a period of 2 minutes) were averaged to obtain a given direct survey measurement, which was surveyed into the same local datum as the tide gauge. We shifted each tide gauge deployment vertically by up to ± 30 cm, in steps of 1 cm, to determine the best-fit vertical shift that minimises the misfit between the tide gauge measurements and all reference water level measurements and/or direct survey measurements made during each deployment period. The best-fit vertical shift determined for each deployment ranged between -6 cm and +8 cm.

The vertically aligned tidal measurements from 2021 to 2022 were used to perform tidal harmonic analysis using the Unified Tidal Analysis and Prediction (UTide) software package[1]. We configured UTide for iteratively re-weighted least-squares to harmonically estimate the automatically selected constituents, including the eight major tidal constituents (M2, S2, K1, O1, N2, K2, P1, Q1). The local tidal model was then used to predict tides over an 18.61-year period from 22 June 2004 to 31 January 2023 to account for the 18.61-year modulation of tides by the nodal tidal cycle. The predicted tides were then used to determine tidal datums for Siloso Point by picking turning points rather than using harmonic constituents to estimate datums due to tidal distortion in shallow waters[2,3] (Figure S2). To derive mean high water spring tide, we

averaged all daily high water levels within ± 3.5 days of each highest spring tide; spring tides were specified as being at least seven days apart. The other tidal datums were similarly calculated (Figure S2).

Text S2. How coral microatolls record RSL

The upward growth of coral microatolls are controlled by the lowest tides. Prolonged subaerial exposure at extremely low water levels causes a diedown, in which the uppermost parts of the coral desiccates and dies down to a roughly uniform elevation known as the highest level of survival (HLS)[4,5] (Figures 2a & 2b). Subsequent upward growth produces a new concentric ring as the coral's highest level of growth (HLG) catches up to the annual lowest tides (or the theoretical HLS). During this time, if the lowest tides rise more quickly than the corals can grow up, the coral's HLG temporarily decouples from sea level, until it is close enough to the theoretical HLS that a transient sea-level lowering causes a new diedown again[5] (Figure 2). Therefore, the pre-diedown HLG provides a filtered record of RSL changes through time; between diedowns, the coral's HLG is limited by its growth rate, rather than sea level[6].

In the years within the 18.61-yrs nodal tidal cycle when the lowest tides get successively lower each year[5,7], a new diedown can be expected to occur each year, producing a cluster of closely-spaced diedowns (Figure 2). The magnitude of a diedown (and thus HLS) can be affected by stochastic non-tidal effects (e.g., oceanographic phenomena like the El Niño-Southern Oscillation and Indian Ocean Dipole, and/or extreme local meteorological events)[5,8]. In contrast, the pre-diedown HLG most closely tracks the non-extreme, astronomically-driven lowest tides superimposed on seasonal oscillations[9] (Figure 2).

We demonstrate how we reconstructed RSL with an example for the youngest SLIP using the outer core of SILO F3 (SILO F3 OUT) (Figures 3b & 5). We subtracted the reference water level of living *Diploastrea heliopora* at Siloso Point (-1.51 m SHD) from the elevation of the top of the core (-1.54 m SHD) to derive the uncorrected RSL of -0.03 m. We subtracted 0.03 m from the RSL to account for a systematic offset in the elevations of living HLG, which were surveyed within a year or two after a diedown (Supporting Text S8). This produced a final corrected RSL of -0.06 m for SILO F3 OUT (Table 2, Supporting Document SI1).

To quantify the total vertical uncertainty (2σ) of the SILO F3 OUT SLIP, we added in quadrature 1) the uncertainty in the offset between the living HLG surveyed and the pre-diedown HLG (± 0.02 m, 2σ; Supporting Text S7); 2) the indicative range uncertainty for *Diploastrea heliopora* (± 0.10 m, 2σ); 3) the uncertainty associated with determining the indicative range (± 0.07 m, 2σ); and 4) the levelling uncertainty (± 0.01 m) – deriving the total vertical RSL uncertainty of ± 0.13 m (2σ) (Supporting Document SI1). Here, the indicative range uncertainty (± 0.10 m, 2σ) is determined from the standard deviation of the living *Diploastrea heliopora* HLG/HLS measured across Kusu and Semakau Islands. The uncertainty in determining the indicative range (± 0.07 m, 2σ) stems from having to relate the living HLG/HLS of *Diploastrea heliopora* to that of *Porites* sp. microatolls to estimate an indicative meaning for living *Diploastrea heliopora* microatolls at Siloso Point (Supporting Text S6 & S6; Supporting Document SI2).

To account for the time it would have taken for the dated sample in SILO F3 OUT to grow upwards by 11 cm from the depth it was sampled to the surface, we applied an age extrapolation to the core top (Table 1, Supporting Text S5), deriving a final age of 633 ± 4 yrs BP, or 1317 ± 4 CE (Table 2).

Text S3. Elevation surveys

Elevation surveys at Siloso Point were conducted on multiple separate field trips between 2020 and 2022 using a total station. To tie the multiple surveys into the same reference frame, we established local temporary benchmarks at the study site. Benchmarks that were common between two successive total station surveys were used as backsights to rotate each survey to the one before it; all surveys were ultimately rotated to the same reference frame as the first survey at Siloso Point.

To relate the relative surveys of the total station to real-world elevations, we surveyed the elevations of several temporary benchmarks (BS5b, BS6b, BS9, BS10, BS11) at Siloso Point, relative to a nearby national geodetic benchmark (VCP 862808) maintained by the Singapore Land Authority (SLA) using a digital level. The national geodetic datum is the Singapore Height Datum (SHD). The digital levelling survey produced a closure error of < 1 mm. Most of the temporary benchmarks (BS5b, BS6b, BS9, BS10) were also surveyed using the total station on the same day. The relative elevations between temporary benchmarks measured using the total station were consistent with that measured using the digital level to < 3 mm. We determined the vertical shift needed to convert elevations in the local total station reference frame at Siloso Point to the SHD by finding the difference between the two for each temporary benchmark, then taking the average difference (Supporting document SI3). Surveys of the living corals at Kusu and Semakau Islands were not tied to the surveys at Siloso Point. The relative surveys at Kusu and Semakau Islands were done using a total station and digital level respectively.

On fossil corals, we surveyed the elevation of the top of each core (at the ring crest) prior to drilling. To georeference the digital surface models created using Structure-from-Motion

photogrammetry, three to five unique features that were identifiable both on the ground and in the photographs of the corals (e.g., screw, edge of a crack) were surveyed on fossil microatolls SILO F1, SILO F15, SILO F18 and SILO F3. The unique features were chosen such that they were spread across the coral surface, rather than being concentrated in one part of the coral.

On each living coral, we surveyed the highest HLG and/or HLS points around the outer living ring, avoiding anomalous depressions in HLG where the coral may have locally died down lower due to non sea-level factors like bioerosion and physical abrasion[10,11].

We constructed an orthomosaic of the reef using drone imagery collected on the day with the lowest tides in 2021 (29 May 2021, with the lowest observed tide of -1.66 m SHD). To georeference the orthomosaic to reflect real-world coordinates, we surveyed ground control points that were identifiable from the drone imagery (e.g., unique features on coral microatolls, line on the pavement) using the GNSS network RTK technique that takes real-time corrections from the Singapore reference network, SiReNT. All measurements were referenced to the SVY21 coordinate system datum.

Text S4. Sample preparation for dating and calculation of U-Th dates

The cores were ultrasonically cleaned in distilled water and oven-dried at 40°C. We then sampled the cores at depth to obtain ~1 cm slices for U-Th dating, avoiding stained or recrystallised segments of the cores. Selected subsamples were further divided into two independent samples to check for reproducibility of the dates (denoted as ‘B1’ and ‘B2’).

All 22 samples were coarsely crushed into smaller, thin segments to pick out ~200 mg of the most pristine-looking pieces for U-Th dating. The remaining pieces were ground to powder using an agate mortar and pestle for powder X-ray Diffraction (XRD) to screen for calcite. The

powdered samples were scanned using a Bruker D2 Phaser diffractometer, which scanned between 20 – 90° 2θ angles with a 0.02 step per second.

The peak diffraction angles of our samples were compared to those of aragonite and calcite references to determine the relative abundance of the calcite and aragonite polymorphs[12,13]. We ran a phase quantification analysis on each sample's 1D peak profile using DIFFRAC.TOPAS V6; the TOPAS software determines the percentage abundance of calcite and aragonite and any other phases present. As small amounts of NaCl and SiO_2 phases were detected in some samples, we refined the relative abundance of calcite in each sample by dividing the relative abundance of calcite by the relative abundance of aragonite and calcite phases combined[14]. All 22 powder XRD samples were determined to have <1% relative abundance of calcite, indicating that there is unlikely to have been diagenetic alteration from secondary calcite in these subsamples[15].

The most pristine pieces selected for U-Th dating were cleaned thrice again using an ultrasonic cleaning machine with milli-Q water and dried at approximately 50°C. For each sample, about 50 mg of the subsample was used for U-Th chemistry. Ultraclean nitric acid was used to dissolve all the samples, followed by the addition of a ^{229}Th-^{233}U-^{236}U mixed spike solution. Organic compounds were removed using a few drops of $HClO_4$. The dried samples were then refluxed at 175°C for two hours, redissolved in 2N ultrapure HCl solution, and co-precipitated with $FeCl_3$ and concentrated NH_4OH. The co-precipitates were redissolved and purified through anion-exchange resin columns to separate and purify uranium and thorium. Finally, U and Th fractions were dissolved in 1% HNO_3 (+0.1% HF) for instrumental analysis. The chemical procedures followed closely the protocols described by Ref.[16] and Ref.[17].

The isotopic compositions of U and Th were measured using a Neptune Plus multi-collector inductively coupled plasma mass spectrometer (MC-ICP-MS) located at the isotope geochemistry laboratory in the Earth Observatory of Singapore (EOS) and Asian School of the Environment (ASE), Nanyang Technological University of Singapore. The SEM peak-jumping technique, improved recently[18,19], was employed for measuring all U and Th isotopes, except ^{238}U and ^{232}Th, on a secondary electron multiplier (SEM) equipped with a retarding potential quadruple lens (RPQ) to enhance abundance sensitivity. The half-lives of ^{234}U and ^{230}Th were obtained from Ref.[18], while the half-life of ^{238}U was acquired from Ref.[20]. The initial $^{230}Th/^{232}Th$ atomic ratio was assumed to be $4.4 \pm 2.2 \times 10^{-6}$ (2σ), and the corrected ^{230}Th ages were computed accordingly. Initial $^{230}Th/^{232}Th$ atomic ratios determined by isochrons from coral microatolls in Sumatra produced the same mean value but with a larger, asymmetrical uncertainty of 4.4 (+6.8 and -2.7) x 10^{-6} (2σ) in linear space (derived from $1.47 \pm 0.95 \times 10^{-6}$ (2σ) in log normal space)[21].

To explore the influence of larger variability than assumed in the $^{230}Th/^{232}Th$ atomic ratio, we conducted a sensitivity test, varying initial $^{230}Th/^{232}Th$ atomic ratios between 0 and 10 x 10^{-6} (Figures S8 and S9). An assumed atomic ratio of 0 simply yields the uncorrected ^{230}Th ages, which are given in Supporting document SI1, whereas ages determined assuming an atomic ratio of 10 x 10^{-6} are given in Table S1. The sensitivity test reveals that the ^{230}Th ages are mostly insensitive to the assumed initial $^{230}Th/^{232}Th$ atomic ratio; the gentle slopes in Figures S8 and S9 indicate little change to the corrected ^{230}Th ages when the assumed initial $^{230}Th/^{232}Th$ atomic ratio is varied. The sample from SILO F18 CEN (S1212) is the most sensitive to the assumed initial $^{230}Th/^{232}Th$ atomic ratio due to its higher ^{232}Th content[22] (Supporting document SI1). Nonetheless, the age of the SILO F18 CEN core top that was independently determined from the coral profile of SILO F18 CEN (which was scaled to the core-top ages of the SILO

F18 IN and SILO F18 OUT cores; Supporting Text S10) matches the absolute ^{230}Th age of the SILO F18 CEN core top (Figure 5). This suggests that given the current morphological constraints of the coral, and under the simple assumption of no large growth-rate changes through the lifetime of the coral, there is no evidence to refute the assumed initial $^{230}Th/^{232}Th$ atomic ratio used. The relative ages of the cores continue to remain in stratigraphic order even at the extreme end of initial $^{230}Th/^{232}Th$ atomic ratios tested – showing the robustness of the ages in the context of elucidating the Late Holocene RSL history at Siloso Point. The U-Th dates' uncertainties are reported at the 2σ level.

Table S1. Mean, mode, 68% and 95% credible range of corrected ^{230}Th ages, assuming $(^{230}Th/^{232}Th)_i$ value of 10×10^{-6}. All corrected ^{230}Th ages are in years before present (BP), where 'present' refers to 1950 CE.

Coral core	Lab ID	Mean	Mode	68% range		95% range	
F1-out	S1199	2650.1	2650	2646.5	2653.8	2642.8	2657.4
F1-out	S1214	2639.2	2639.3	2635.5	2642.9	2631.9	2646.5
F1-in	S1200	2795.8	2795.9	2792.3	2799.4	2788.7	2802.9
F2-out	S1201	2155.9	2156	2152.9	2158.9	2149.9	2161.9
F2-in	S1202	2748.5	2748.6	2744.2	2752.9	2739.8	2757.3
F2-in	S1215	2747.2	2747.2	2743.5	2750.8	2739.8	2754.6
F3-out	S1203	699.58	699.61	697.88	701.28	696.18	702.99
F3-out	S1216	704.25	704.26	702.97	705.54	701.7	706.84
F3-in	S1204	905.36	905.42	903.66	907.04	901.97	908.74
F3-in	S1217	903.84	903.86	902.41	905.28	900.98	906.72
F5-B	S1205	2304.5	2304.6	2301.3	2307.7	2298.2	2310.9
F6-B	S1206	2849.9	2850.4	2846.1	2853.8	2842.3	2857.6
F7-B	S1207	2875.8	2876.1	2871.7	2880	2867.5	2884.2
F15-out	S1208	1793.6	1793.4	1791	1796.1	1788.5	1798.7
F15-mid	S1209	1942.3	1942.4	1939.5	1945.2	1936.6	1948
F15-in	S1210	1965.6	1965.6	1962.4	1968.7	1959.3	1971.8
F18-out	S1211	1691.9	1691.7	1689.2	1694.5	1686.6	1697.1
F18-out	S1219	1691.6	1691.4	1688.7	1694.4	1685.9	1697.2
F18-in	S1213	1838.9	1838.9	1836.2	1841.5	1833.5	1844.2
F18-in	S1221	1835.7	1836	1832.3	1839.1	1829	1842.5
F18-center	S1212	1881.1	1881	1878.3	1883.9	1875.4	1886.7
F18-center	S1220	1879.9	1879.9	1876.6	1883.2	1873.3	1886.5

Text S5. Age extrapolation to core top

As the samples for dating were subsampled at depth, we needed to make an age extrapolation for the time it would have taken for the coral at depth to have grown to the surface[11]. The extrapolated age for the top of the core was derived from Equation 8:

$$t' = t - \frac{d \cos \theta}{r} \tag{8}$$

Where *t'* is the extrapolated age of the core top, *t* is the ^{230}Th age of the dated sample (sampled at depth within the core), *d* is the sample depth, θ is the declination of the growth direction from the long axis of the core (i.e., acute angle from the vertical) (Figures S4 and S5), and *r* is the coral growth rate. For samples with replicate ^{230}Th dates (i.e., B1 and B2 subsamples), the age extrapolation was made to the weighted mean age of both samples and the associated standard error (Table 1, Supplementary document SI1).

If the growth direction in the core had been perfectly horizontal and the growth bands were perfectly parallel to the core along the drilling direction (which was generally vertical), the age of the sample at depth would be identical to the age of the microatoll surface.

Uncertainties in the extrapolated age of the top of the core were propagated using a Monte Carlo approach. We assumed a uniform distribution for the growth angle (θ), whose minimum and maximum bounds are core-specific and are determined by visual inspection of the maximum and minimum growth angles observed within the core (Figure S4); and a uniform distribution for sample depth (*d*), governed by the measured sample thickness. For SILO F15, whose growth directions are variable within the core, we assumed a conversative bound for the growth angle between 0° and 90°. We assigned a normal distribution $N(4,1^2)$ mm/yr for possible growth rates (*r*), centred on the growth rates of *Diploastrea heliopora* reported in literature (2 – 6 mm/yr)[23–25]. We excluded all random samples with < 0.5 mm/yr growth rates

as such slow growth rates are unlikely, and because in reality, there is no physical meaning to negative growth rates. The 95th percent credible interval derived from the highest density region of the probability distribution of each core top age is used as the width of each of the SLIPs and marine limiting data (Figure S5).

Text S6. Calculating a theoretical level for HLG of Diploastrea at Siloso Point

There were no living *Diploastrea heliopora* coral microatolls at Siloso Point. To calculate RSL, we calculated a theoretical level for HLG of living *Diploastrea heliopora* at Siloso Point by extrapolation of the elevational relationship between living *Diploastrea heliopora* and *Porites* sp. microatolls at the nearby Kusu and Semakau Islands (Supporting Document SI2). Microatolls of different genus have been documented to grow at different elevations due to differential tolerance to various environmental parameters, including the degree of subaerial exposure[26–28]. We assume that the inter-genus difference in the indicative meaning at any given site is systematic, as it is related to the intrinsic tolerance of the respective coral genus to environmental factors, and corals located at the same site should experience similar environmental parameters.

We first calculated the elevation of each living *Diploastrea* microatoll relative to *Porites* microatolls at each site (Equations 3 & 4). For each living *Diploastrea* microatoll,

$$E_{i,Diplo-Porites,Kusu} = E_{i,Diplo,Kusu} - E_{Porites\ wtd\ mean,Kusu} \quad (3)$$

$$E_{i,Diplo-Porites,SMK} = E_{i,Diplo,SMK} - E_{Porites\ wtd\ mean,SMK} \quad (4)$$

where $E_{i,Diplo}$ represents the elevation of the ith living *Diploastrea* microatoll, $E_{Porites\ wtd\ mean}$ represents the weighted mean elevation of the *Porites* microatolls at the same site as the *Diploastrea* microatoll and $E_{i,Diplo-Porites}$ represents the elevation of the

Diploastrea microatoll relative to the weighted mean elevation of the *Porites* microatolls at the same site.

The weighted mean elevation of *Porites* microatolls at each site was used as a common datum to combine the indicative meaning of the five *Diploastrea heliopora* microatolls across the Kusu Island and Semakau Island sites (Figure S7). The difference in elevation between *Diploastrea heliopora* microatolls and *Porites* sp. microatolls were similar between Kusu and Semakau Islands (Figure S7), supporting our assumption that there is a systematic bias between *Diploastrea heliopora* microatolls and *Porites* sp. microatolls at a given site, regardless of the hydrogeomorphic setting.

We calculated the weighted mean elevation of the *Diploastrea heliopora* microatolls (relative to the weighted mean elevation of *Porites* microatolls across all sites), using the adjusted elevations, E_i, from Equations 3 and 4.

$$E_{Diplo-Porites\,wtd\,mean,all\,sites} = \frac{\sum_{i=1}^{n}\left(\frac{E_{i,Diplo-Porites}}{\sigma_{i,Diplo-Porites}^{2}}\right)}{\sum_{i=1}^{n}\left(\frac{1}{\sigma_{i,Diplo-Porites}^{2}}\right)} \quad (5)$$

We then added the above adjustment to the weighted mean elevation of *Porites* microatolls at Siloso Point to determine a theoretical level for the HLG of living *Diploastrea* microatolls at Siloso Point (Equation 6).

$$E_{dl} = E_{Porites\,wtd\,mean,SILO} + E_{Diplo-Porites\,wtd\,mean,all\,sites} \quad (6)$$

Text S7. Propagation of vertical uncertainties in RSL reconstructions

To determine the vertical uncertainty in the relative sea-level reconstructions, we added in quadrature six uncertainty terms (Equation 2, Table S2):

Table S2. Sources of vertical uncertainties (2σ) in RSL reconstructions.

No.	Uncertainty Source	Uncertainty (m)
1	Standard error of weighted mean elevation of modern *Porites* sp. microatolls at Siloso Point	± 0.038
2	Standard error of weighted mean elevation of modern *Porites* sp. microatolls at Kusu	± 0.028
3	Standard error of weighted mean elevation of modern *Porites* sp. microatolls at Semakau	± 0.046
4	Standard error of weighted mean elevation of modern *Diploastrea heliopora* microatolls across both the Kusu and Semakau sites	± 0.031
5	Standard deviation of the elevations of modern *Diploastrea heliopora* microatolls across both the Kusu and Semakau sites	± 0.102
6	Levelling uncertainty	± 0.006

Uncertainty terms 1-4 are associated with determining the theoretical elevation of living *Diploastrea heliopora* microatolls at Siloso Point, uncertainty term 5 represents the indicative range uncertainty of *Diploastrea heliopora* microatolls at Siloso Point, and uncertainty term 6 is the uncertainty associated with measurements of each fossil coral sample. The levelling uncertainty is the 2σ elevation range determined from repeat measurements of backsights across independent total station surveys.

For all calculations of the standard error of the weighted mean (uncertainty terms 1-4), we corrected for over- and under-dispersion (Equation 7).

$$\hat{\sigma} = \sqrt{\frac{1}{\sum_{i=1}^{n} \sigma_i^{-2}} \cdot \frac{1}{n-1} \cdot \sum_{i=1}^{n} \frac{(x_i - \bar{x})^2}{\sigma_i^2}} \qquad (7)$$

where $\hat{\sigma}$ is the standard error of the weighted mean, corrected for over- and under-dispersion, σ_i and x_i are the standard deviation and mean elevation of measurements on the ith coral and n is the total number of corals. $\bar{x}$ is the inverse-variance weighted mean HLG/HLS elevation of the living *Diploastrea heliopora* coral microatolls across the Kusu and Semakau Islands.

Text S8. Additional corrections to RSL determinations

All living microatolls that were surveyed in this study had experienced recent diedowns (within a year or two prior to the time of the surveys), so the living HLG surveyed were similar to the

HLS of the recent diedowns. Strictly speaking, the elevations of the fossil microatoll ring crests should be compared to the HLG just before the diedown, rather than the HLS after a diedown. We subtracted an additional 3 ± 2 cm (2σ) from the RSL of all reconstructed data points to account for this offset in the living HLG surveys by quantifying the largest difference between the mean pre-diedown HLG and the mean post-diedown HLG/HLS of the living corals that had surveys of both the pre-diedown HLG and post-diedown HLG/HLS (n = 15).

Comparison of the morphologies of SILO F15 and SILO F18 suggests SILO F15 is heavily eroded, unlike SILO F18 that is well-preserved. The HLG of rings 4 through 1 on SILO F15 generally decreases outwards by up to ~0.08 m, in contrast to the HLG of rings 3 and 2 on SILO F18 that increases outwards by ~0.16 m (Figures 3, 4B & 5), even though these should have been coeval. Areas of locally aggravated erosion are indicated by potholes that are not concentric around the coral (Figure 3C). Additionally, the corallites on SILO F15 are oriented nearly horizontally (Figure S10), which indicates that what is preserved and visible on SILO F15 today was close to the base of the coral where the growth direction would have been horizontal; this suggests that substantial thickness of coral material above it has been eroded away. SILO F15 also lacks any visible overgrowth segments, which are found only on the surface of microatolls and are visible in SILO F1, SILO F18 and SILO F3. One possible interpretation that may explain this difference in elevation and preservation between SILO F15 and SILO F18, despite their close proximity, is that the diedown event that killed R1 on SILO F15 entirely only killed the upper part of the coeval ring on SILO F18 (now hidden beneath the overgrowth on R1 of SILO F18). Subsequently, SILO F18 continued to grow outwards and upwards (and grew the overgrowth on R1 of SILO F18), while SILO F15 was dead and therefore could not build upwards and instead suffered erosion. The differential erosion of

coeval microatolls (and indeed across a given fossil coral microatoll) is also possible (e.g., Ref.[29]).

We added a uni-directional uncertainty to account for the erosion on SILO F15. To quantify the degree of erosion on SILO-F15, we aligned the scaled cross-sectional profiles of SILO F15 and SILO F18 using the common diedown (Figures 3 and 4, Figure S11), then calculated the difference in the surface elevation of SILO-F15 and SILO-F18 during periods of overlap. We applied the 95th percentile confidence interval of this difference as an uncertainty in the upward direction as erosion would have lowered the RSL recorded by SILO F15. We added the midpoint of this range (+ 0.082 m) to the RSL and added half of this range (± 0.107 m, 2σ) in quadrature with the existing uncertainty for each of the SILO F15 SLIPs. This is a conservative estimate of the amount of erosion as SILO F18 has some out-of-sequence overgrowth obscuring the rings beneath, so the true differences between coeval rings of SILO F18 and SILO F15 are less than what is currently captured by the elevation differences.

Text S9. Structure-from-Motion photogrammetry

Photographs of the fossil corals SILO F1, SILO F18 and SILO F3 were taken at the lowest of tides when the fossil corals were fully subaerially exposed. A digital surface model was also produced for the eroded SILO F15 on the day with the lowest predicted tide between 2020 and 2022 (-1.85 m SHD predicted for the Tanjong Pagar tide gauge on 17 June 2022) when the ring crests of the coral were exposed. However, even at the lowest tides observed on this day (-1.58 m SHD), parts of the low grooves between rings were under water. Due to refraction across the air-water interface, the apparent depth in parts of the DEM that are submerged under water would be shallower than the true depth of the coral surface[30]. We selected the transect of SILO F15 such that only negligible parts of the profile are submerged (Figure S11). All digital surface

models were georeferenced using unique features (e.g., screw, end of a crack) that were surveyed on the coral microatolls using the total station.

Text S10. Scaling of coral cross-sectional profiles

To support the inferences of RSL change, we translated the horizontal distance along each ith coral microatoll cross-sectional profile (x_i) into age estimates ($t_{i,scaled}$) to investigate the sea-level tendencies during each coral's lifetime (Equations 9 to 14):

$$x_{i,interval} = x_{i,out} - x_{i,in} \tag{9}$$

$$x_{i,mid} = \frac{x_{i,out} + x_{i,in}}{2} \tag{10}$$

$$t_{i,interval} = t_{i,in} - t_{i,out} \tag{11}$$

$$\overline{t_i} = \frac{t_{i,out} + t_{i,in}}{2} \tag{12}$$

$$d_i = x_i - x_{mid} \tag{13}$$

$$t_{i,scaled} = \overline{t_i} + (\frac{d_i}{x_{i,interval}} \times t_{i,interval}) \tag{14}$$

Where $x_{interval}$ and $t_{interval}$ are the respective horizontal distance and age interval between the inner and outer cores, $x_{i,mid}$ is the midpoint between the inner and outer cores and $\overline{t_i}$ is the mean age of the inner and outer cores. To derive the $t_{interval}$ and $\overline{t_i}$, we used the central value ($t_{i,out}$ and $t_{i,in}$) of the 95% credible interval (highest density region) of the age distributions of the inner and outer cores respectively. d_i refers to the distance of a given point along the

transect from the midpoint between the inner and outer cores. We note that the profiles were only scaled horizontally, with no changes made to the elevations along the profile.

The scaled profiles of SILO F15 and SILO F18 were than translated horizontally to align the two corals to a common diedown (Figure 4B), while maintaining that the position of each dated core lies within its calculated age at the core top, derived from its ^{230}Th date(s) (Supporting text S5).

We calculated the horizontal extension rate for each coral based on the distance-to-age ratios of each pair of cores (Equation 15).

$$g_i = \frac{x_{i,interval}}{t_{i,interval}} \qquad (15)$$

The horizontal extension rates (g_i) of the coral microatolls derived from our cross-sectional profiles and core ages range between 4 and 8 mm/yr, in close agreement with the linear extension rates of *Diploastrea heliopora* reported in literature, of 2 – 6 mm/yr[23–25].

Text S11. Updated Late Holocene RSL database for the Sunda Shelf

An updated RSL database was produced for the interior of the Sunda Shelf, where there is minimal RSL variability from glacial isostatic adjustment (GIA) during the Late Holocene. The database was produced following the HOLocene SEA-level variability (HOLSEA) database protocol[31]. RSL data were only considered valid if the indicators had a known age, geographic location, tidal elevation and indicative meaning; the absence of any one of these criteria renders a RSL data point rejected. A summary of the indicative meanings applied to the RSL data are provided in Tables S3 and S4. A binary classification was applied to categorise the RSL data as low or high quality. Samples were classified as low quality if they

were susceptible to unquantifiable errors in the age, elevation or indicative meaning (Table S5)[32].

All radiocarbon ages were calibrated using the latest IntCal20[33] and Marine20[34] calibrations in OxCal 4.4[35]. Values for the marine reservoir correction (ΔR) were taken from the original publications where available, and otherwise extracted from the Marine20 ΔR database[36]. All U-Th ages were screened for high detrital content (^{232}Th concentrations ranging from 20-90 ppb; ^{230}Th/^{232}Th activity ratios <100) and anomalous δ^{234}U values (greater than ± 5 ‰ deviation from the modern seawater value of 145 ‰) – all samples passed the screening criteria.

Table S3. Summary of the indicative meanings that were applied to samples, where local surveys of the indicative meaning were absent. LAT: lowest astronomical tide; MTL: mean tide level; MHWN: mean high water neaps; MHHW: mean higher high water; HAT: highest astronomical tide; RWL: reference water level; IR: indicative range.

**Indicator**	**Evidence**	**RWL**	**IR**
**Sea-level index points**			
Emerged oysters	Zone of oysters attached to rock surfaces[37–39]	$\frac{LAT+HAT}{2}$	$\frac{HAT-LAT}{2}$
Intertidal deposit	Stratigraphic interpretation (unit between underlying paleosol and overlying marine mud)	$\frac{LAT+HAT}{2}$	$\frac{HAT-LAT}{2}$
Shore platform	Abraded coral reef; inner margin of the shore platform forms near MHHW, with lower margin forming near breaking depth and MLLW[40], which we interpret conservatively at LAT	$\frac{LAT+MHHW}{2}$	$\frac{MHHW-LAT}{2}$
Mangrove sediment	Mangrove sediments with dominance of brackish diatoms[41]; mangrove wood[42]	$\frac{LAT+MTL}{2}$	$\frac{MTL-LAT}{2}$
Mangrove sediment with *Nypa* associations	Leaf remains of *Nypa fruticans* (Nipah palm) found with piece of wood with seagrass remains indicating reach of seawater[43]; *Nypa fruticans* are found between MHWN and HAT[44,45]	$\frac{MHWN+HAT}{2}$	$\frac{HAT-MHWN}{2}$
**Marine limiting**			
Coral (possibly ponded)	Coral that could have been growing at significant depths[46] but could also have been growing up to MHWN in a pond that did not drain fully at low tide due to the presence of a	MHWN	< MHWN

	topographically high sill (rocks) that ponds water landwards of it[47].		
Coralline algae	Crusts of calcareous red algae that can live in intertidal and subtidal environments, which are prone to desiccation[48–50] but which can live above mean sea level depending on exposure to wave splash[51]	MHHW	< MHHW
**Terrestrial limiting**			
Freshwater peat/ undifferentiated peat	Freshwater peat characterised by freshwater plant macrofossils, or peat with no evidence to differentiate between mangrove and freshwater environments[52,53]	MTL	> MTL

Table S4. Table of locally surveyed indicative meanings reported by the authors in original references. RWL: reference water level; IR: indicative range; NLSD: National Land Survey Datum of Peninsula Malaysia; msl: mean sea level.

Reference	Indicator	Evidence	RWL	IR uncertainty
Tam et al. (2018)	Cyeraceae swamp	pollen assemblage	0.2 m NLSD	± 0.2 m
Tam et al. (2018)	Mid mangrove	pollen assemblage	0.4 m NLSD	± 0.15 m
Tam et al. (2018)	Back mangrove	pollen assemblage	0.5 m NLSD	± 0.1 m
Tam et al. (2018)	*Nypa* swamp	pollen assemblage	0.5 m NLSD	± 0.2 m
Tam et al. (2018)	Gelam forest	pollen assemblage	0.95 m NLSD	± 0.3 m
Zhang et al. (2021)	Transition zone between mangrove and estuarine swamp	pollen assemblage	0.9 m msl	± 0.5 m
Zhang et al. (2021)	Estuarine swamp	pollen assemblage	1.2 m msl	± 0.35 m
Zhang et al. (2021)	Transition zone between estuarine swamp and upland vegetation	pollen assemblage	1.6 m msl	± 0.3 m
Wan et al. (2020)	*Porites* sp. coral microatoll	concentric rings characteristic of microatolls	23.7 cm above lowest predicted tide in 2012	± 28.7 cm

Table S5. Qualitative criteria used to categorise samples as low quality.

**Age errors**	**Elevation errors**	**Indicative meaning errors**
Age may have been contaminated by younger roots	Sample may have been post-depositionally lowered; post-depositional lowering unaccounted for or uncertain	No evidence/ microfossil analyses to confirm provenance of sediments
Age of coral is a maximum age for the shore platform (abraded coral reef)		
Age of sample is a maximum age as the sample may not be in-situ		

References

1. Codiga, D. Unified tidal analysis and prediction using the UTide Matlab functions. *Technical Report 2011-01* (2011) doi:10.13140/RG.2.1.3761.2008.
2. Pugh, D. & Woodworth, P. *Sea-Level Science: Understanding Tides, Surges, Tsunamis and Mean Sea-Level Changes*. (Cambridge University Press, 2014). doi:10.1017/CBO9781139235778.
3. Woodworth, P. L. Differences between mean tide level and mean sea level. *J Geod* **91**, 69–90 (2017).
4. Woodroffe, C. & McLean, R. Microatolls and recent sea level change on coral atolls. *Nature* **344**, 531–534 (1990).
5. Meltzner, A. J. *et al.* Half-metre sea-level fluctuations on centennial timescales from mid-Holocene corals of Southeast Asia. *Nat Commun* **8**, 14387 (2017).
6. Smithers, S. G. & Woodroffe, C. D. Coral microatolls and 20th century sea level in the eastern Indian Ocean. *Earth and Planetary Science Letters* **191**, 173–184 (2001).

7. Haigh, I. D., Eliot, M. & Pattiaratchi, C. Global influences of the 18.61 year nodal cycle and 8.85 year cycle of lunar perigee on high tidal levels. *Journal of Geophysical Research: Oceans* **116**, (2011).
8. Philibosian, B. *et al.* Rupture and variable coupling behavior of the Mentawai segment of the Sunda megathrust during the supercycle culmination of 1797 to 1833. *J. Geophys. Res. Solid Earth* **119**, 7258–7287 (2014).
9. Meltzner, A. J. *et al.* Coral evidence for earthquake recurrence and an A.D. 1390–1455 cluster at the south end of the 2004 Aceh–Andaman rupture. *J. Geophys. Res.* **115**, B10402 (2010).
10. Smithers, S. G. & Woodroffe, C. D. Microatolls as sea-level indicators on a mid-ocean atoll. *Marine Geology* **168**, 61–78 (2000).
11. Wan, J. X. W. *et al.* Relative sea-level stability and the radiocarbon marine reservoir correction at Natuna Island, Indonesia, since 6400 yr BP. *Marine Geology* **430**, 106342 (2020).
12. Farfan, G. A. *et al.* Crystallographic and chemical signatures in coral skeletal aragonite. *Coral Reefs* **41**, 19–34 (2022).
13. Rahman, M. A., Halfar, J. & Shinjo, R. X-Ray Diffraction Is a Promising Tool to Characterize Coral Skeletons. *AMPC* **03**, 120–125 (2013).
14. Sayani, H. R. *et al.* Effects of diagenesis on paleoclimate reconstructions from modern and young fossil corals. *Geochimica et Cosmochimica Acta* **75**, 6361–6373 (2011).
15. Smodej, J. *et al.* Two-dimensional X-ray diffraction as a tool for the rapid, nondestructive detection of low calcite quantities in aragonitic corals. *Geochem Geophys Geosyst* **16**, 3778–3788 (2015).

16. Edwards, R. L., Chen, J. H. & Wasserburg, G. J. 238U234U230Th232Th systematics and the precise measurement of time over the past 500,000 years. *Earth and Planetary Science Letters* **81**, 175–192 (1987).

17. Shen, C.-C. *et al.* High-precision and high-resolution carbonate 230Th dating by MC-ICP-MS with SEM protocols. *Geochimica et Cosmochimica Acta* **99**, 71–86 (2012).

18. Cheng, H. *et al.* Improvements in 230Th dating, 230Th and 234U half-life values, and U–Th isotopic measurements by multi-collector inductively coupled plasma mass spectrometry. *Earth and Planetary Science Letters* **371–372**, 82–91 (2013).

19. Chiang, H.-W., Lu, Y., Wang, X., Lin, K. & Liu, X. Optimizing MC-ICP-MS with SEM protocols for determination of U and Th isotope ratios and 230Th ages in carbonates. *Quaternary Geochronology* **50**, 75–90 (2019).

20. Jaffey, A. H., Flynn, K. F., Glendenin, L. E., Bentley, W. C. & Essling, A. M. Precision Measurement of Half-Lives and Specific Activities of U 235 and U 238. *Phys. Rev. C* **4**, 1889–1906 (1971).

21. Chiang, H.-W. *et al.* Investigating spatio-temporal variability of initial 230Th/232Th in intertidal corals. *Quaternary Science Reviews* **307**, 108005 (2023).

22. Edwards, R. L., Gallup, C. D. & Cheng, H. Uranium-series Dating of Marine and Lacustrine Carbonates. (2003).

23. Cantin, N. E., Cohen, A. L., Karnauskas, K. B., Tarrant, A. M. & McCorkle, D. C. Ocean Warming Slows Coral Growth in the Central Red Sea. *Science* **329**, 322–325 (2010).

24. Schuhmacher, H., Loch, K., Loch, W. & See, W. R. The aftermath of coral bleaching on a Maldivian reef—a quantitative study. *Facies* **51**, 80–92 (2005).

25. Watanabe, T. *et al.* Oxygen isotope systematics in Diploastrea heliopora: new coral archive of tropical paleoclimate. *Geochimica et Cosmochimica Acta* **67**, 1349–1358 (2003).

26. Meltzner, A. J. & Woodroffe, C. D. Coral microatolls. in *Handbook of Sea-Level Research* 125–145 (John Wiley & Sons, Ltd, 2015). doi:10.1002/9781118452547.ch8.
27. Scoffin, T. P., Brown, B. E., Dunne, R. P. & Tissier, M. D. A. L. The Controls on Growth Form of Intertidal Massive Corals, Phuket, South Thailand. *PALAIOS* **12**, 237 (1997).
28. Smithers, S. Microatoll. in *Encyclopedia of Modern Coral Reefs* 691–696 (Springer, Dordrecht, 2011). doi:10.1007/978-90-481-2639-2_111.
29. Meltzner, A. J. *et al.* Persistent termini of 2004- and 2005-like ruptures of the Sunda megathrust. *Journal of Geophysical Research: Solid Earth* **117**, B04405 (2012).
30. Cao, B., Deng, R. & Zhu, S. Universal algorithm for water depth refraction correction in through-water stereo remote sensing. *International Journal of Applied Earth Observation and Geoinformation* **91**, 102108 (2020).
31. Khan, N. S. *et al.* Inception of a global atlas of sea levels since the Last Glacial Maximum. *Quaternary Science Reviews* **220**, 359–371 (2019).
32. Tan, F. *et al.* Holocene relative sea-level histories of far-field islands in the mid-Pacific. *Quaternary Science Reviews* **310**, 107995 (2023).
33. Reimer, P. J. *et al.* The IntCal20 Northern Hemisphere Radiocarbon Age Calibration Curve (0–55 cal kBP). *Radiocarbon* **62**, 725–757 (2020).
34. Heaton, T. J. *et al.* Marine20—The Marine Radiocarbon Age Calibration Curve (0–55,000 cal BP). *Radiocarbon* **62**, 779–820 (2020).
35. Ramsey, C. B. Bayesian Analysis of Radiocarbon Dates. *Radiocarbon* **51**, 337–360 (2009).
36. Reimer, P. J. & Reimer, R. W. A marine reservoir correction database and on-line interface. Radiocarbon (2001).
37. Foster, B. A. The Barnacles of Fiji, with Observations on the Ecology of Barnacles on Tropical Shores. *Pacific Science* **28**, 35–56 (1974).

38. Lewis, S. E. *et al.* Rapid relative sea-level fall along north-eastern Australia between 1200 and 800cal.yrBP: An appraisal of the oyster evidence. *Marine Geology* **370**, 20–30 (2015).
39. Tjia, H. D. Sea-level changes in the tectonically stable Malay-Thai Peninsula. *Quaternary International* **31**, 95–101 (1996).
40. Rovere, A. *et al.* The analysis of Last Interglacial (MIS 5e) relative sea-level indicators: Reconstructing sea-level in a warmer world. *Earth-Science Reviews* **159**, 404–427 (2016).
41. Kamaludin, H., Akmal, S., Minerals & Geoscience Department Malaysia, Zong, Y., & Department of Earth Sciences, University of Hong Kong. Late Holocene relative low sea level at Merang, Terengganu. *BGSM* **62**, 23–29 (2016).
42. Geyh, M. A., Streif, H. & Kudrass, H.-R. Sea-level changes during the late Pleistocene and Holocene in the Strait of Malacca. *Nature* **278**, 441–443 (1979).
43. Hesp, P., Chang, C. H., Hilton, M., Chou, L. & Turner, I. M. A first tentative Holocene sea-level curve for Singapore. *Journal of Coastal Research* **14**, 308–314 (1998).
44. Tam, C.-Y. *et al.* A below-the-present late Holocene relative sea level and the glacial isostatic adjustment during the Holocene in the Malay Peninsula. *Quaternary Science Reviews* **201**, 206–222 (2018).
45. Zhang, Y. *et al.* The middle-to-late Holocene relative sea-level history, highstand and levering effect on the east coast of Malay Peninsula. *Global and Planetary Change* **196**, 103369 (2021).
46. Hibbert, F. D. *et al.* Coral indicators of past sea-level change: A global repository of U-series dated benchmarks. *Quaternary Science Reviews* **145**, 1–56 (2016).

47. Scoffin, T. P., Stoddart, D. R. & Rosen, B. R. The Nature and Significance of Microatolls. *Philosophical transactions of the Royal Society of London* **284**, 99–122 (1978).

48. Kayanne, H. Deposition of Calcium Carbonate into Holocene Reefs and its Relationato Sea-level Rise and Atmospheric CO,. in *Proceedings of the Seventh International Coral Reef Symposium* (Guam, 1992).

49. Steneck, R. S. The Ecology of Coralline Algal Crusts: Convergent Patterns and Adaptative Strategies. *Annu. Rev. Ecol. Syst.* **17**, 273–303 (1986).

50. Steneck, R. S., Macintyre, I. G. & Reid, R. P. A unique algal ridge system in the Exuma Cays, Bahamas. *Coral Reefs* **16**, 29–37 (1997).

51. Pirazzoli & Montaggioni. Holocene sea-level changes in French Polynesia. *Palaeogeography, Palaeoclimatology, Palaeoecology* **68**, 153–175 (1988).

52. Khan, N. S., Vane, C. H., Engelhart, S. E., Kendrick, C. & Horton, B. P. The application of δ13C, TOC and C/N geochemistry of mangrove sediments to reconstruct Holocene paleoenvironments and relative sea levels, Puerto Rico. *Marine Geology* **415**, 105963 (2019).

53. Waycott, M. *et al.* Vulnerability of mangroves, seagrasses and intertidal flats in the tropical Pacific to climate change. in *Vulnerability of Tropical Pacific Fisheries and Aquaculture to Climate Change* (eds. Bell, J. D., Johnson, J. E. & Hobday, A. J.) (2011).

54. OBIS. Ocean Biodiversity Information System. (2023).

Supporting figures

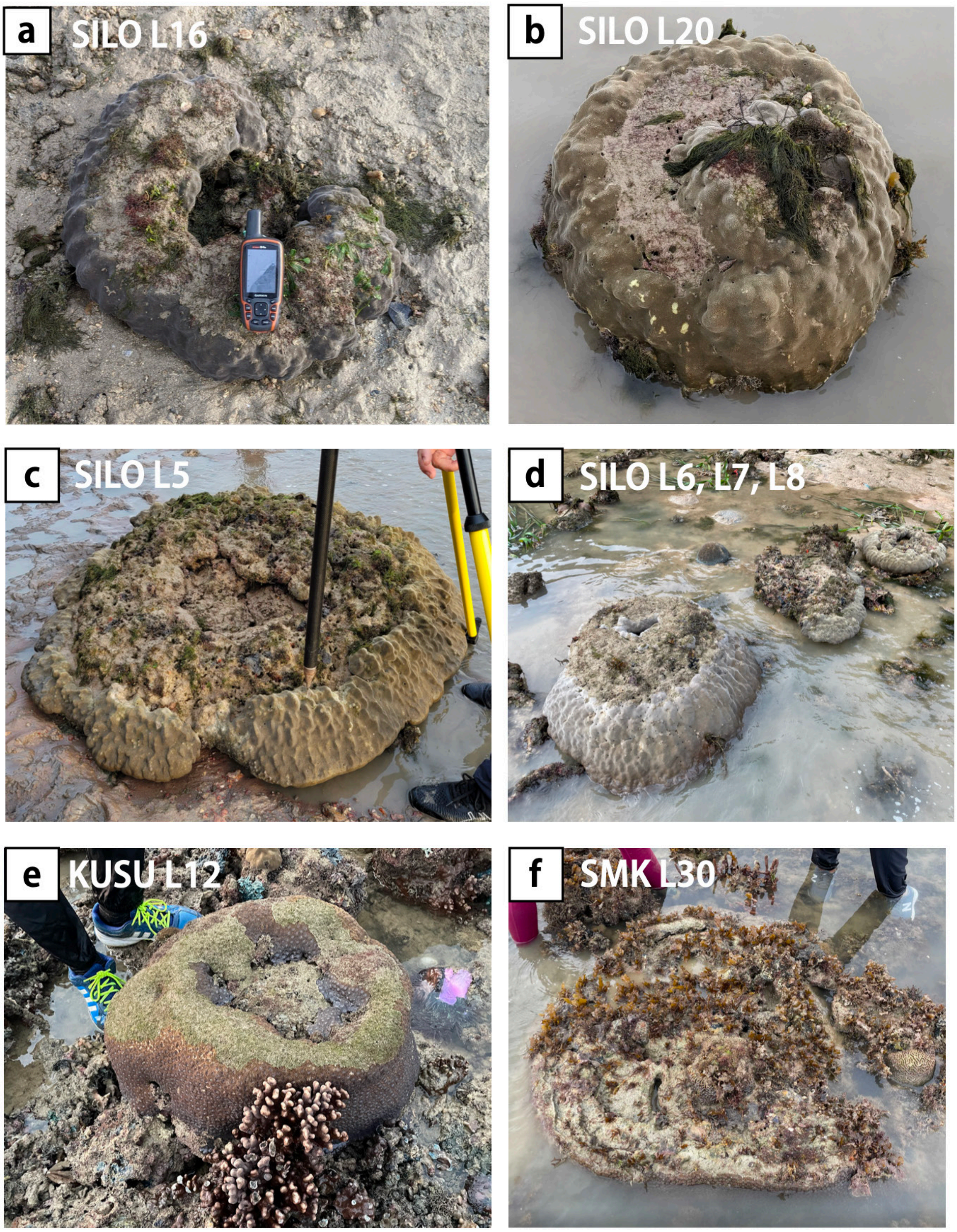

Figure S1. Example photographs of living microatolls in this study. (a-d) Living *Porites* sp. microatolls at Siloso Point, Sentosa. Living *Diploastrea heliopora* microatoll at (e) Kusu Island and (f) Semakau Island.

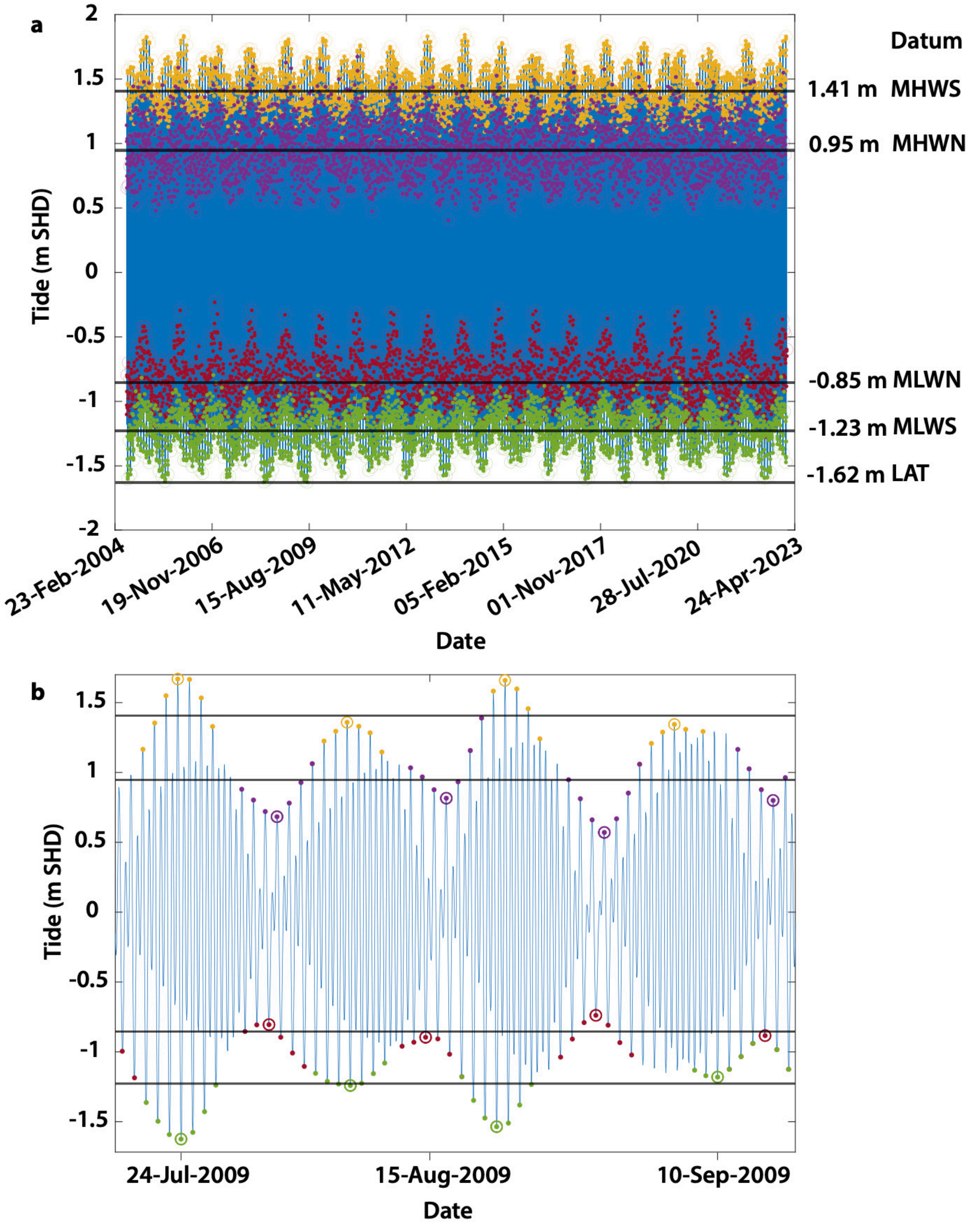

Figure S2. Predicted tides (blue lines) and tidal datums for Siloso Point. Tides were generated using the local tide model that was trained with a year of tide gauge data at Siloso Point (from 2021 to 2022). (a) Predicted tides over the full 18.61-year period. (b) Predicted tides over three spring-neap cycles. Hollow circles indicate the highest and lowest spring and neap tides. Filled dots indicate the high and low waters used to derive the tidal datums: yellow (spring high tides); purple (neap high tides); red (neap low tides); green (spring low tides).

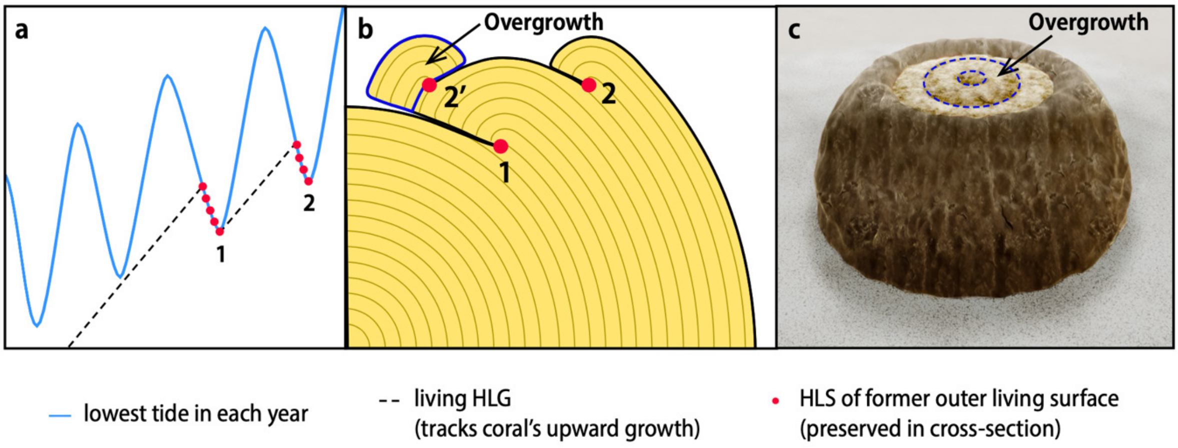

Figure S3. Coral microatoll schematic showing the development of out-of-sequence overgrowth in (b) cross section (radial profile) and its (c) 3D morphology under rising RSL (a). Each cluster of diedowns (red dots) in panel a is grouped collectively into a single red dot in panel b for clarity. In panel b, we show every 3rd annual band for clarity. RSL: relative sea level; HLS: highest level of survival; HLG: highest level of growth.

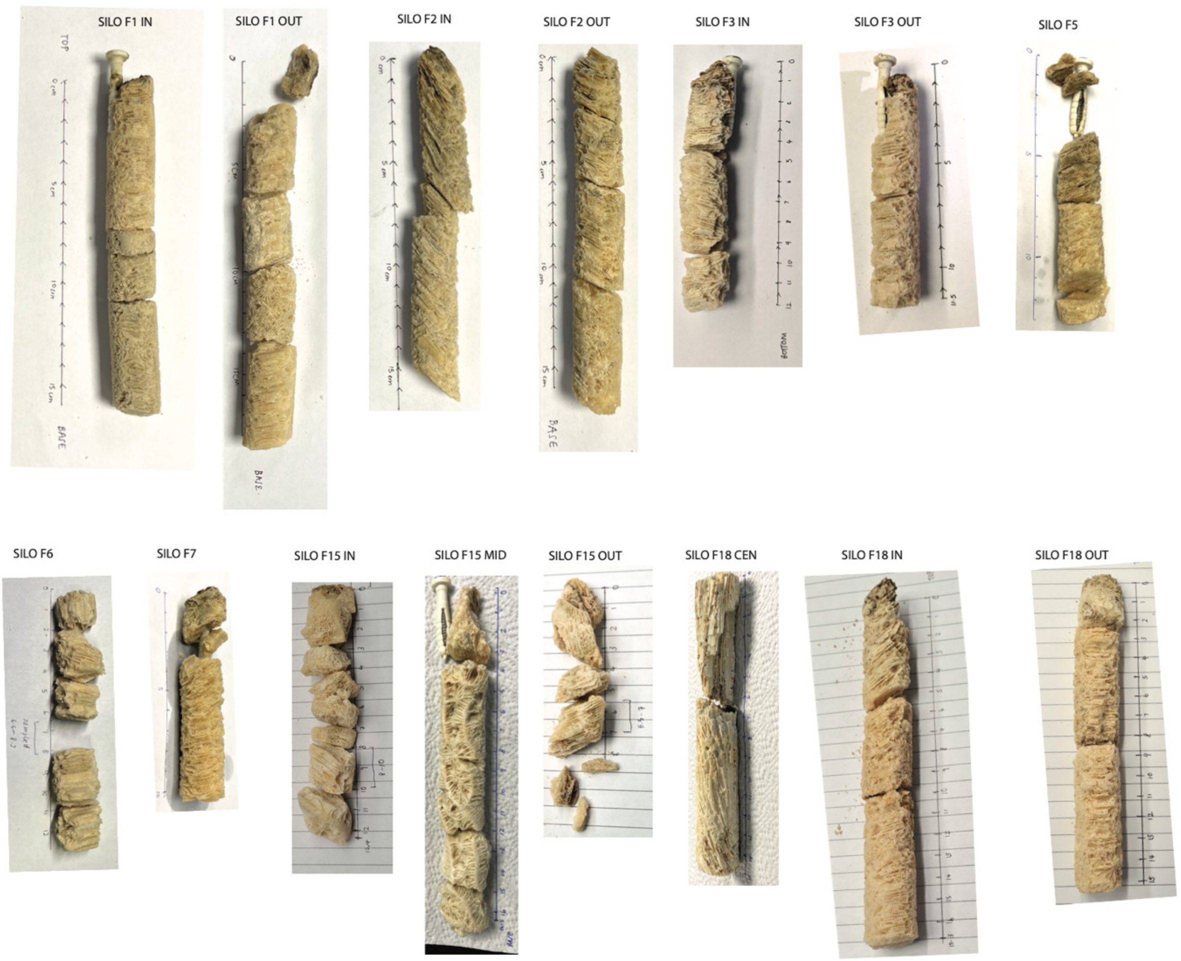

Figure S4. Photographs of all coral cores drilled. Growth direction is parallel to the columella, which are separated by adjoining septa. All cores are oriented vertically, with the top being the surface of the fossil coral.

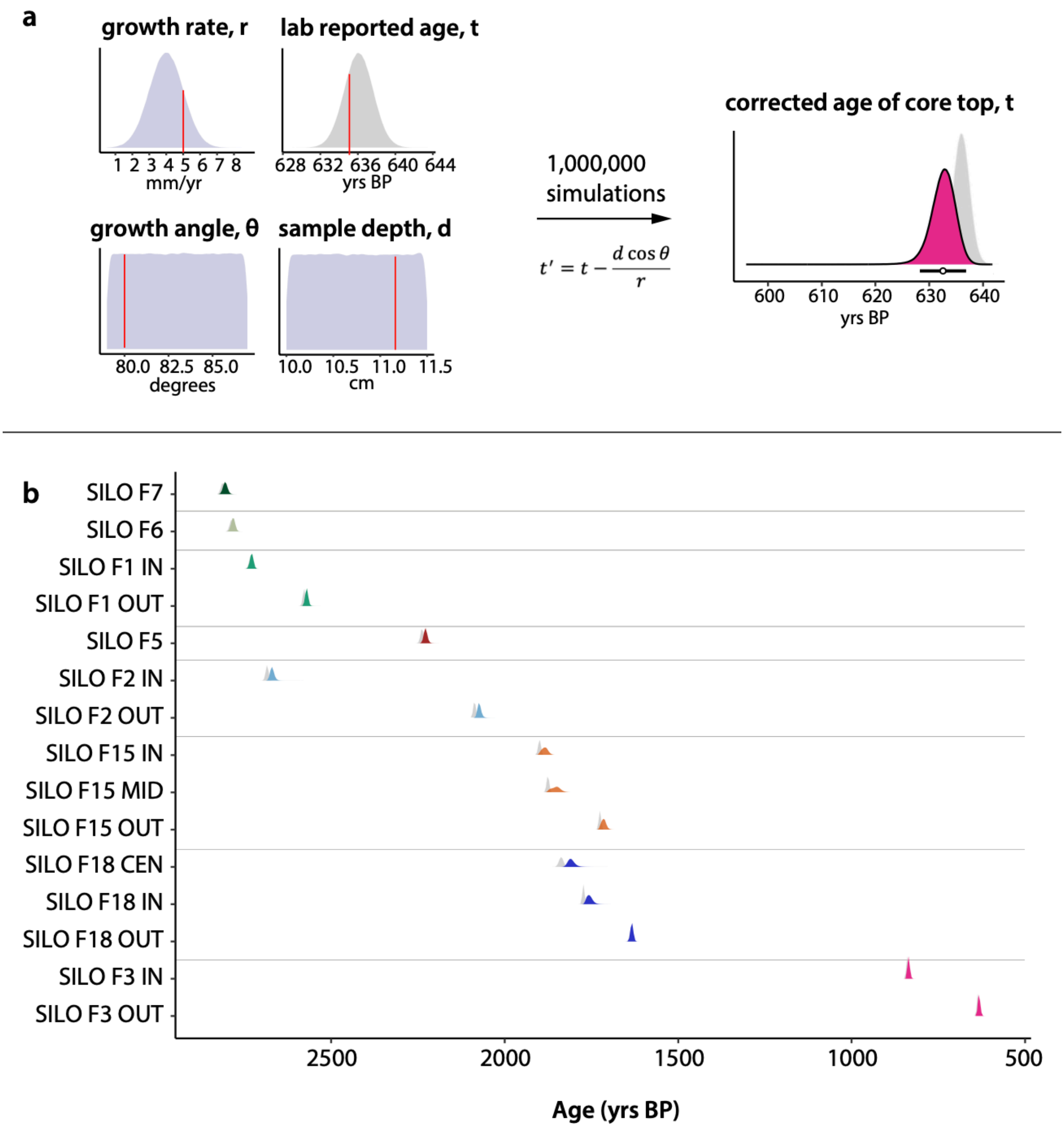

Figure S5. Modelled extrapolated ages for the core tops. (a) Example of Monte Carlo simulation to derive the extrapolated age of the core top of SILO F3 OUT. Each time, a single value (red vertical lines) is sampled from the distributions of the four input parameters to derive a singular value for the final extrapolated age of the core top, t; this is repeated a million times to derive the probability distribution of the age of the core top (pink). The horizontal bar and circle beneath the probability distribution indicates the 95th percent credible interval and mean, derived from highest density region of the core top age. (b) ^{230}Th ages of dated samples taken at depth within each core (grey) and the associated extrapolated age for the top of the core (coloured by coral). For samples with replicate ^{230}Th ages, the grey probability distributions are based on the weighted average of the respective B1 and B2 subsamples (Table 1, Supplementary document SI1).

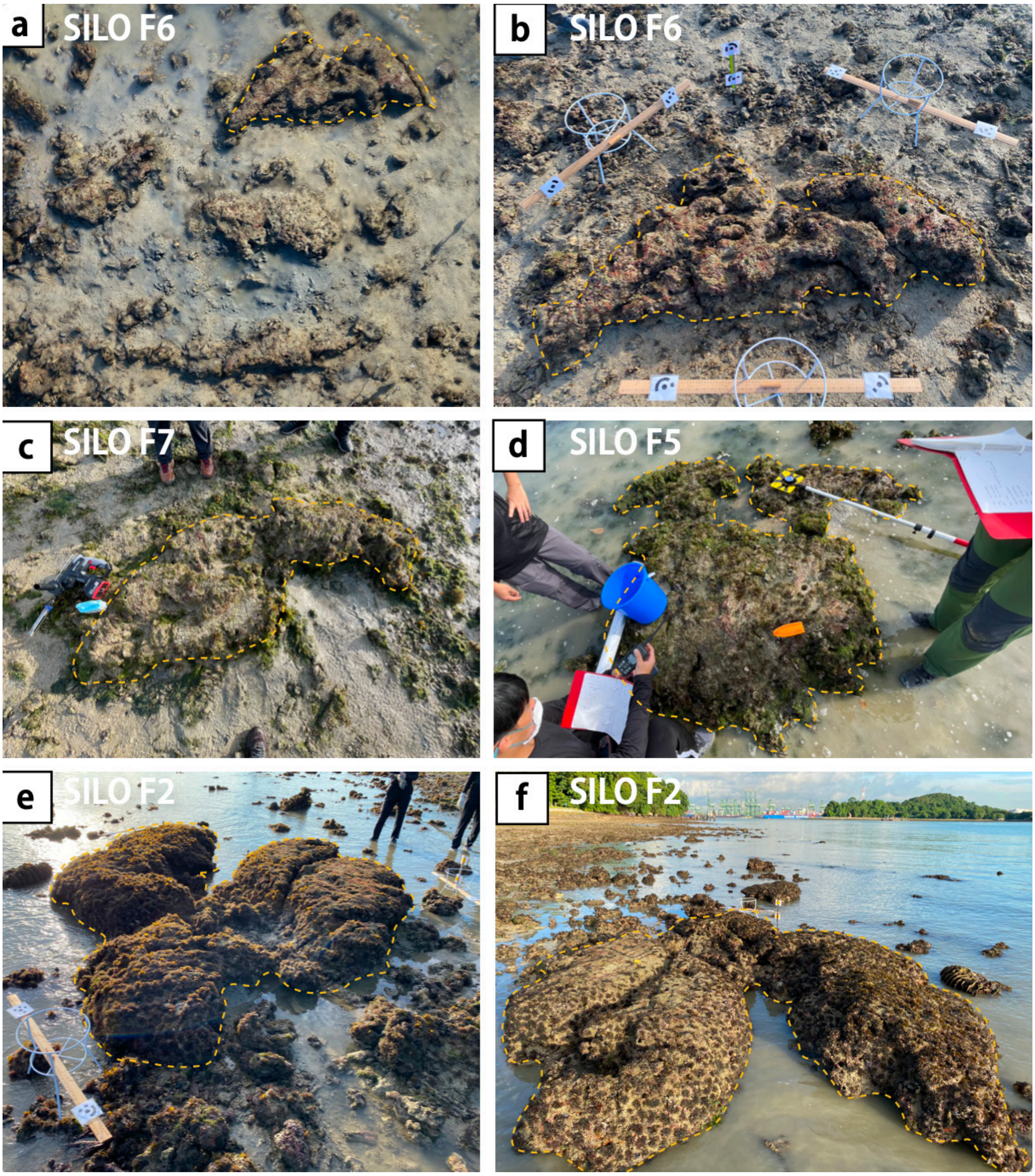

Figure S6. Field photographs of fossil corals without clear concentric-ringed structures. SILO F6 (a-b), SILO F7 (c), SILO F5 (d) and SILO F2 (e-f). Bottom row: SILO F2 viewed from the west (e) and east (f). Yellow dashed lines mark the boundaries of the fossil corals.

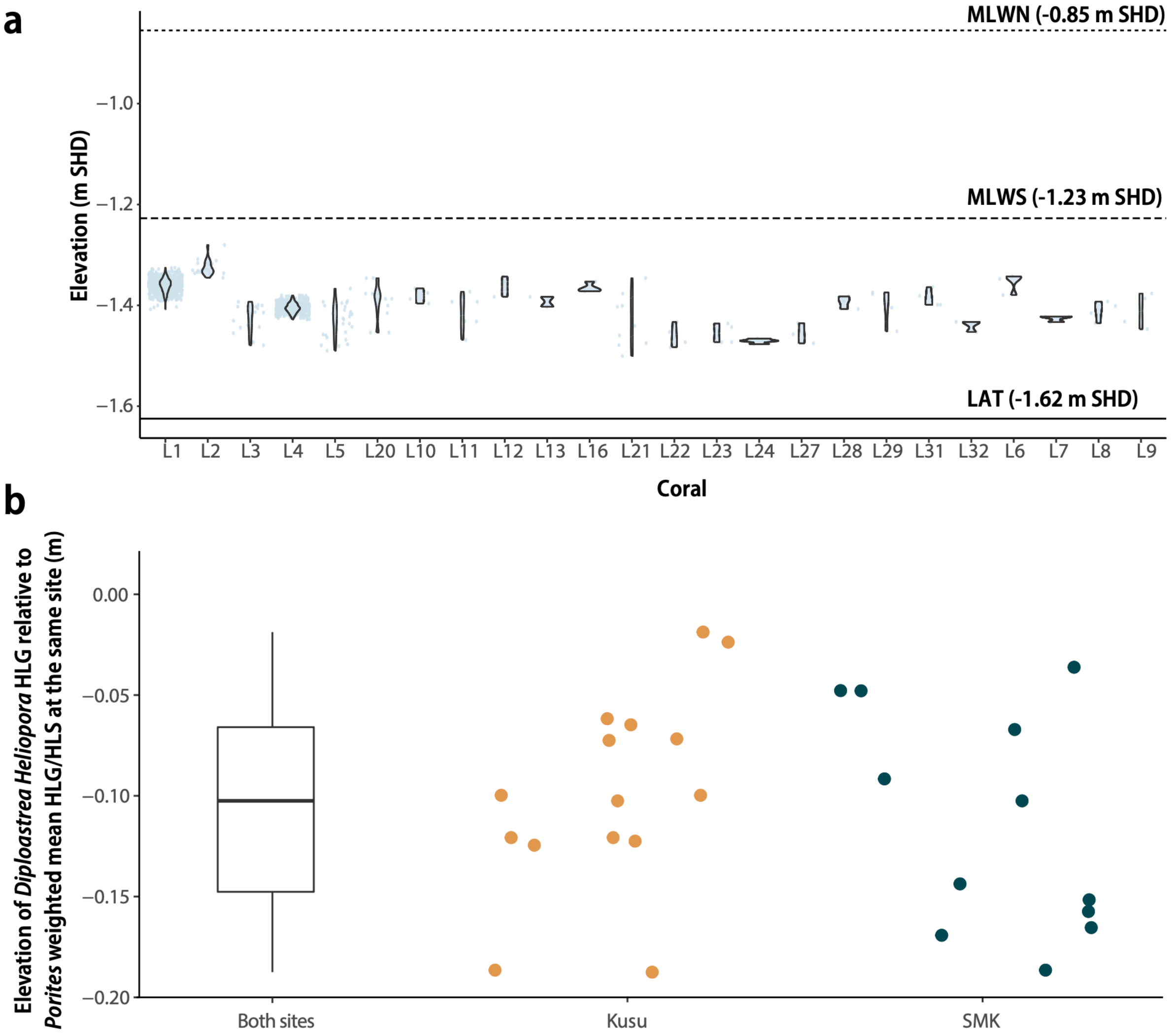

Figure S7. Indicative ranges of living coral microatolls in Singapore. (a) Violin plot showing the HLG elevation range of each living *Porites* sp. microatoll at Siloso Point, Sentosa, relative to MLWS and LAT. (b) Elevation of the HLG/HLS range of living *Diploastrea heliopora* microatolls at the Kusu and Semakau sites, relative to the weighted mean elevation of living *Porites* sp. microatolls at each of these sites. Every point indicates a unique measurement of HLG/HLS. HLG: highest level of growth; HLS: highest level of survival; MLWN: mean low water neaps; MLWS: mean low water springs; LAT: lowest astronomical tide.

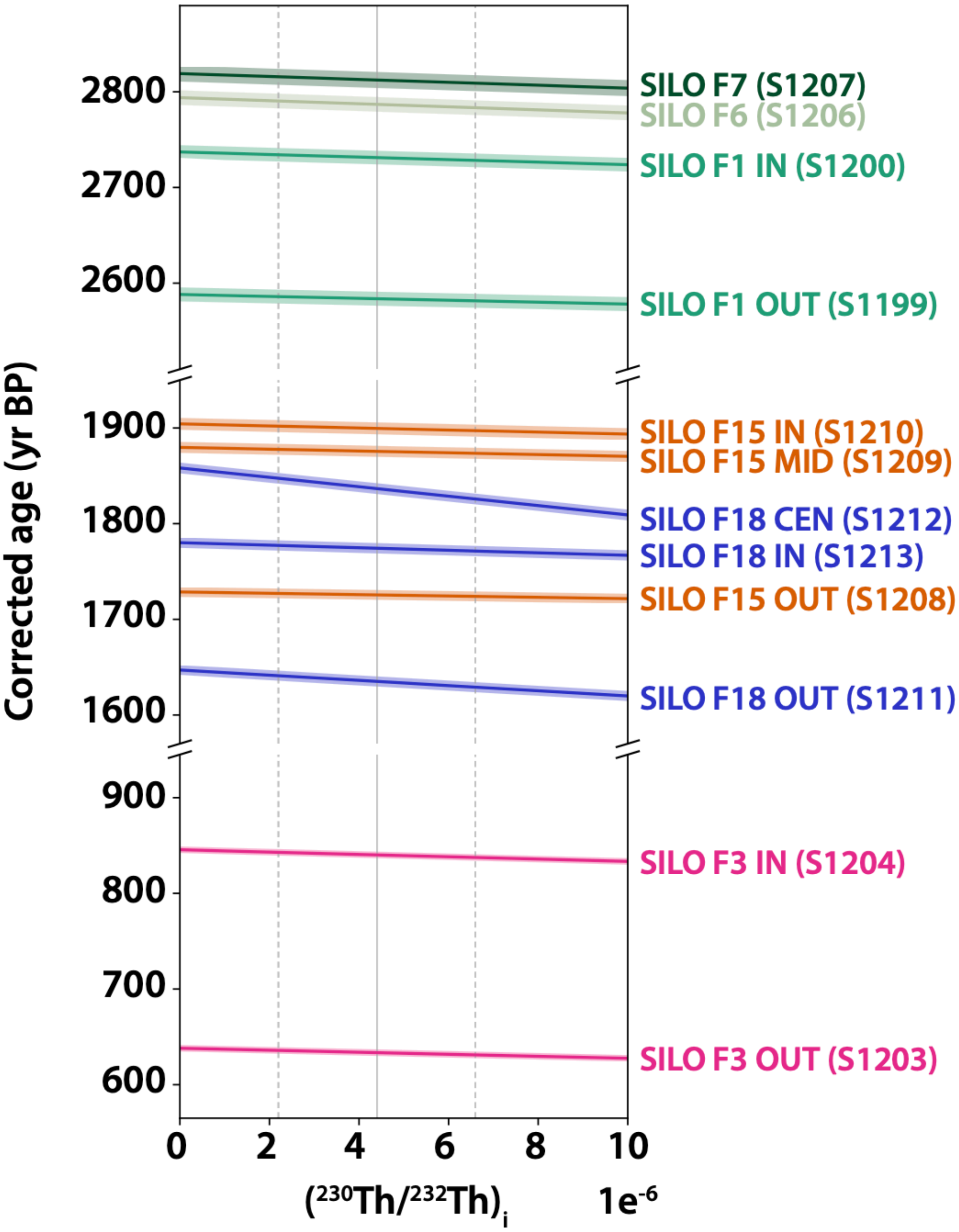

Figure S8. Sensitivity of coral ^{230}Th ages to the initial ^{230}Th/^{232}Th atomic ratios assumed in the correction for initial detrital Th. Shown here are corals that are used as sea-level index points in this study. The uncertainty band in the ages for each sample are the 2σ uncertainties associated with the analytical uncertainties in determining the uncorrected ^{230}Th ages (Supporting document SI1). The solid and dashed vertical lines indicate, respectively, the mean and 2σ bounds of the assumed ^{230}Th/^{232}Th atomic ratio used for the RSL reconstructions in this study: $4.4 \pm 2.2 \times 10^{-6}$.

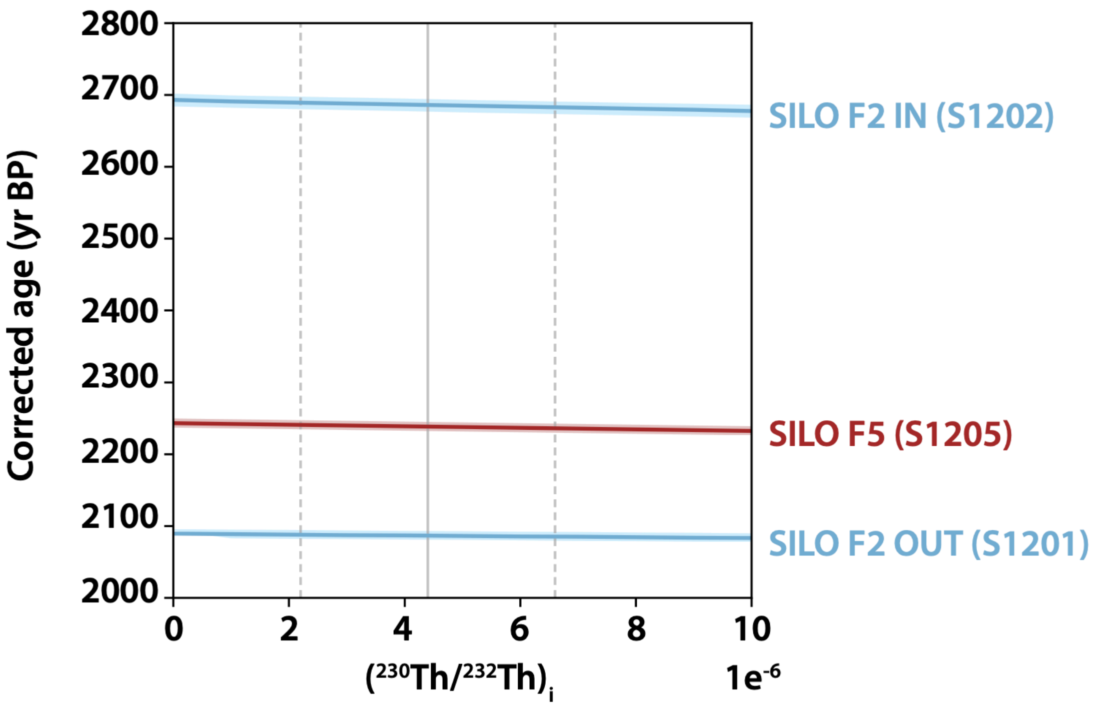

Figure S9. Sensitivity of coral ^{230}Th ages to the initial $^{230}Th/^{232}Th$ atomic ratios assumed in the correction for initial detrital Th. Shown here are corals that are used as marine limiting data in this study. The uncertainty band in the ages for each sample are the 2σ uncertainties associated with the analytical uncertainties in determining the uncorrected ^{230}Th ages (Supporting document SI1). The solid and dashed vertical lines indicate, respectively, the mean and 2σ bounds of the assumed $^{230}Th/^{232}Th$ atomic ratio used for the RSL reconstructions in this study: $4.4 \pm 2.2 \times 10^{-6}$.

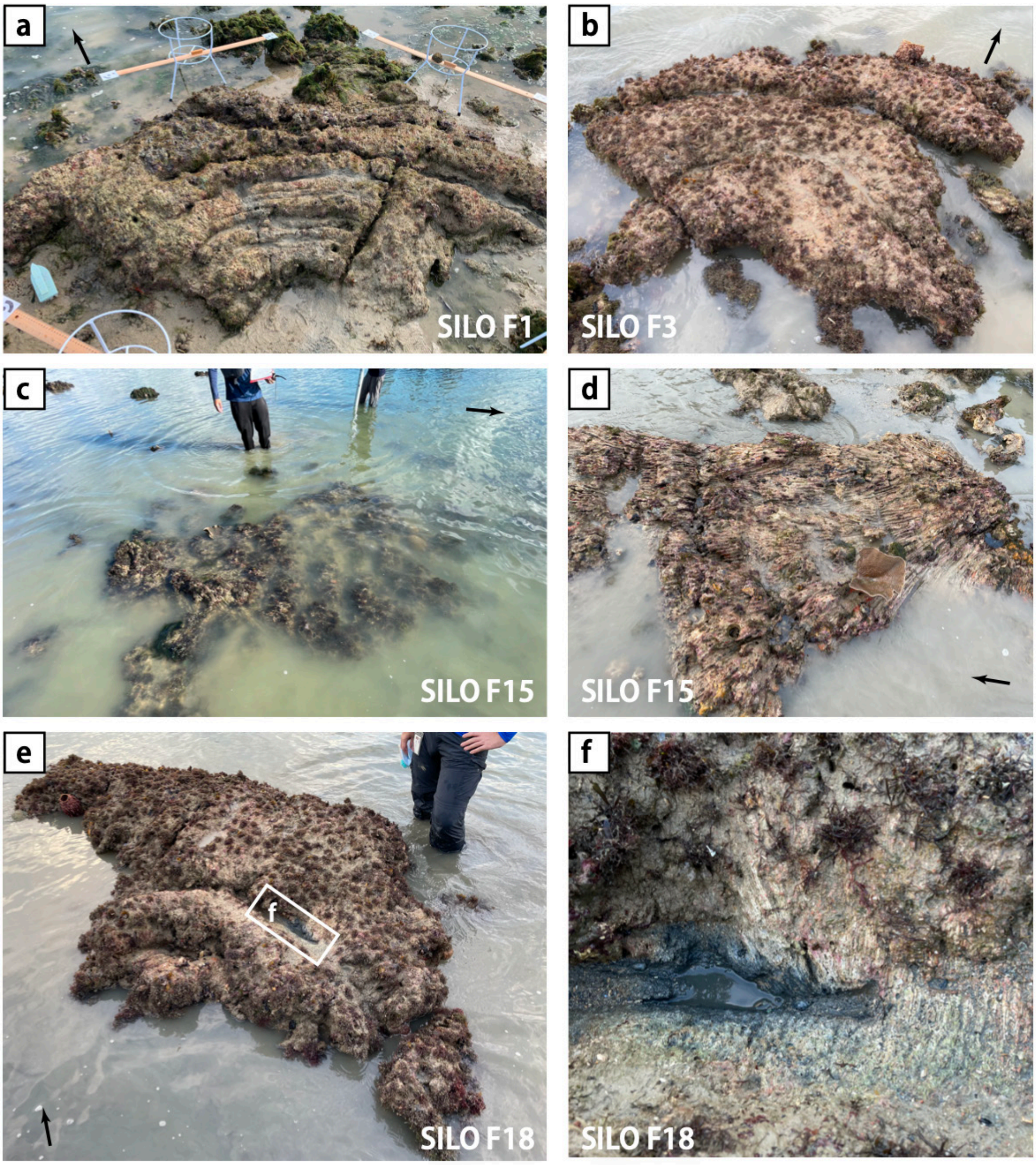

Figure S10. Field photographs of fossil *Diploastrea heliopora* coral microatolls (a) SILO F1, (b) SILO F3, (c-d) SILO F15, and (e-f) SILO F18.

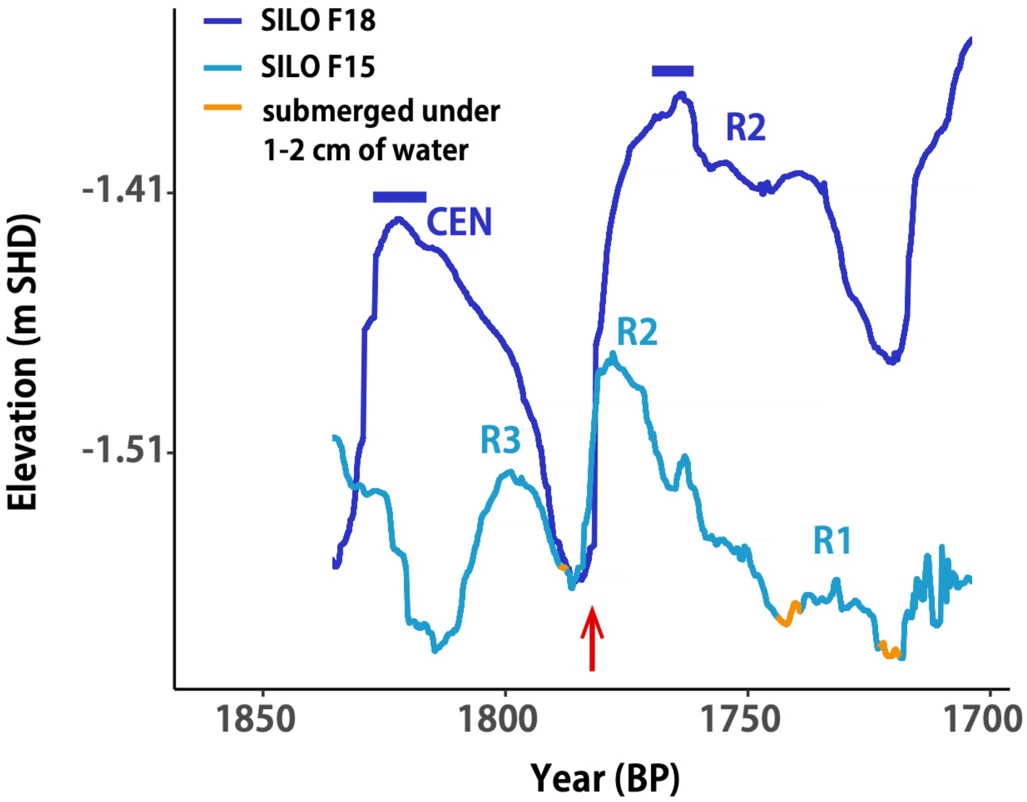

Figure S11. Coeval parts of the SILO F15 and SILO F18 radial cross-sectional profiles, through transects C-C' (SILO F15) and D-D' (SILO F18) (see Figure 3). Red arrow indicates common diedown used to align both cross-sectional profiles.

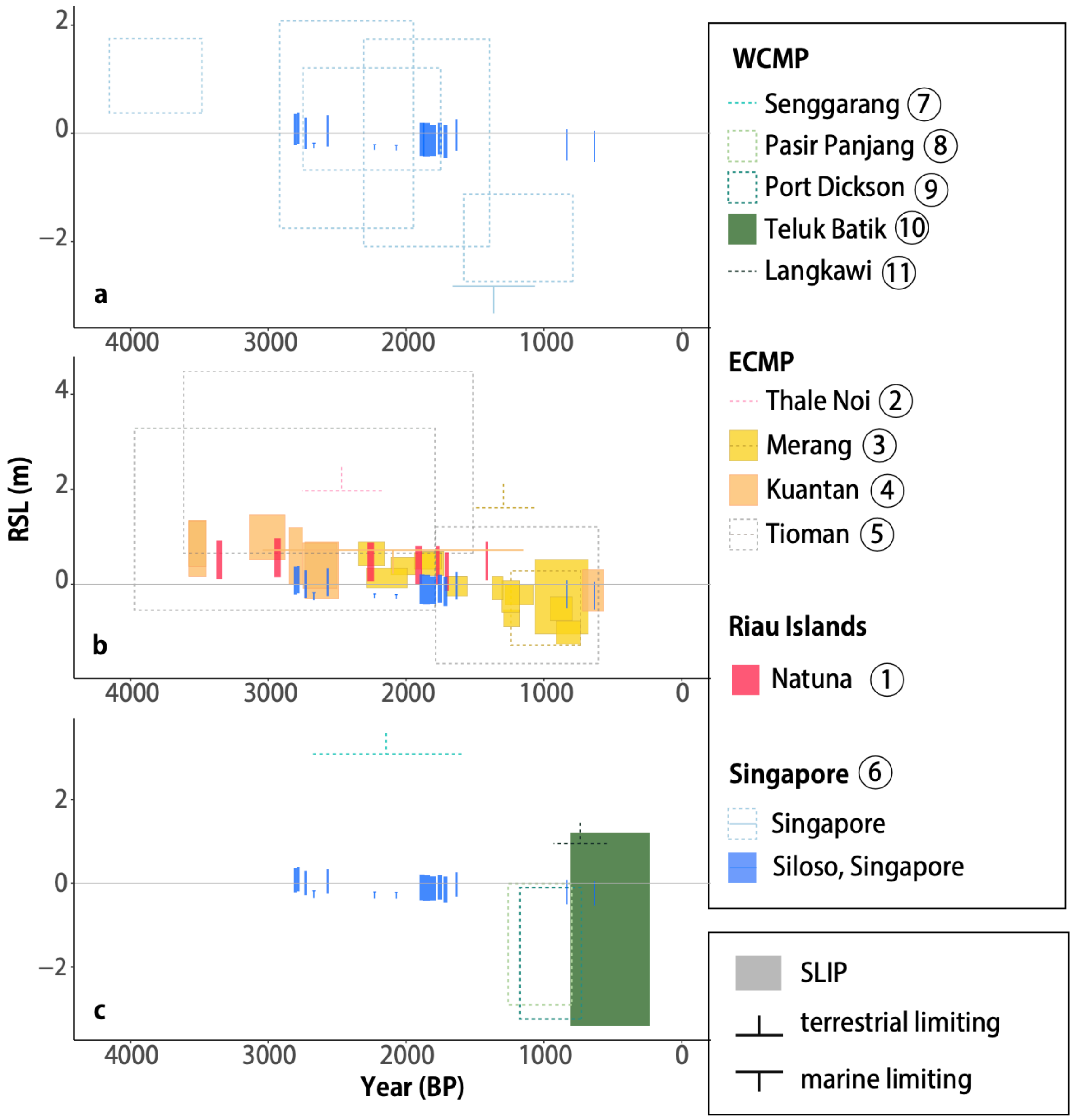

Figure S12. Comparison of the Siloso Point RSL record to published Late Holocene RSL data from the Sunda Shelf, assuming a conservative indicative meaning for the Siloso Point coral microatolls. Late Holocene RSL for Siloso Point, Sentosa, Singapore, compared to data from (a) elsewhere in Singapore (site 6; Figure 7); (b) East Coast Malay Peninsula (ECMP) and Riau Islands (sites 1-5; Figure 7); and (c) West Coast Malay Peninsula (WCMP) (sites 7-11; Figure 7). The Siloso Point RSL data are calculated assuming an indicative meaning of lowest astronomical tide (LAT) to midway between mean low water neaps (MLWN) and mean low water springs (MLWS). Dashed lines: low quality data; solid fill: high quality data. The horizontal line of marine limiting data is plotted at the bottom of the RSL uncertainty and indicate that RSL could have been anywhere at or above the horizontal line, vice versa for terrestrial limiting data, whose horizontal line is plotted at the top of the RSL uncertainty and indicate RSL is at or below it. The vertical ticks in the limiting data are purely symbolic and do not represent the magnitude of RSL uncertainty. RSL: relative sea level; SLIP: sea-level index point.

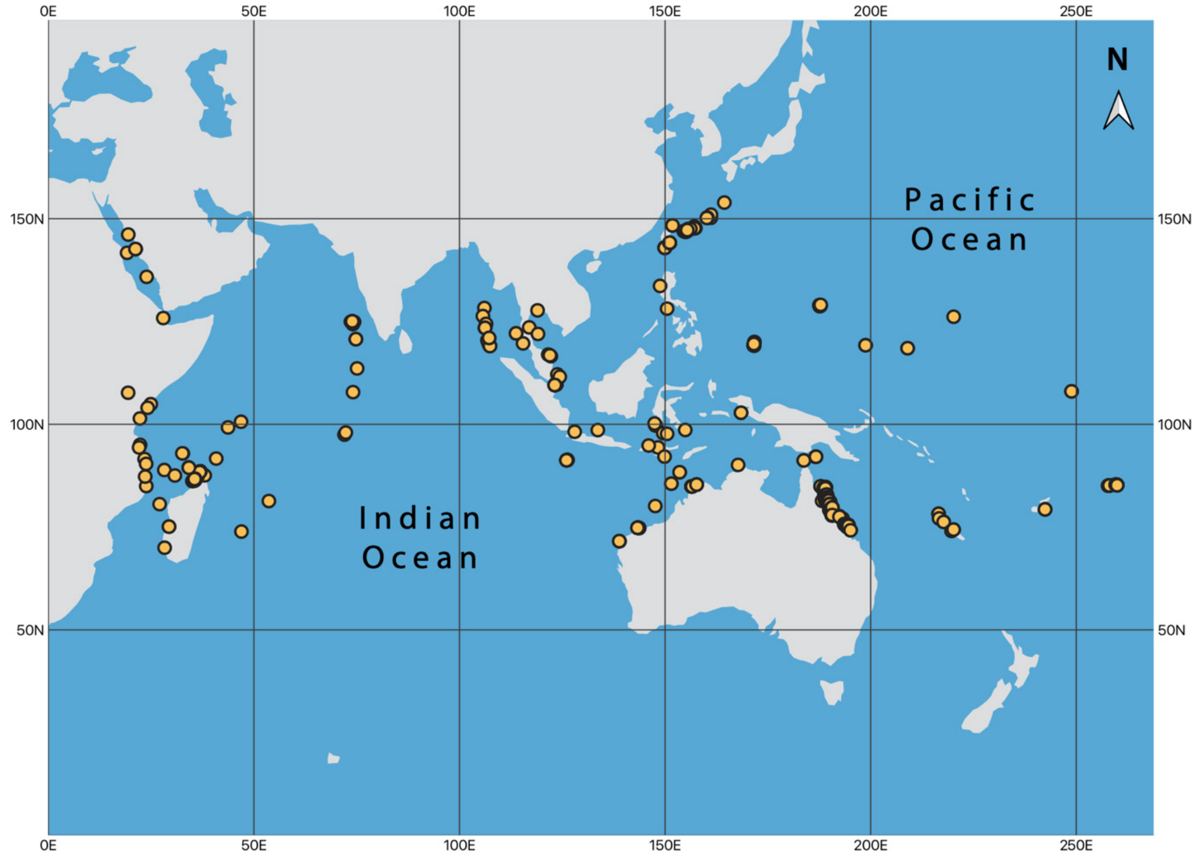

Figure S13. Documented occurrences of *Diploastrea heliopora* corals in the world [54].
